# Resource-efficient fault-tolerant one-way quantum repeater with code concatenation

**DOI:** 10.1038/s41534-023-00792-8

**Published:** 2023-12-12

**Authors:** Kah Jen Wo, Guus Avis, Filip Rozpędek, Maria Flors Mor-Ruiz, Gregor Pieplow, Tim Schröder, Liang Jiang, Anders S. Sørensen, Johannes Borregaard

**Affiliations:** 1 QuTech, Delft University of Technology, Lorentzweg 1, 2628 CJ Delft, The Netherlands; 2 Centre for Quantum Technologies, National University of Singapore, Queenstown, 117543 Singapore; 3 Quantum Computer Science, EEMCS, Delft University of Technology, Lorentzweg 1, 2628 CJ Delft, The Netherlands; 4 Kavli Institute of Nanoscience, Delft University of Technology, Lorentzweg 1, 2628 CJ Delft, The Netherlands; 5 College of Information and Computer Sciences, University of Massachusetts Amherst, Amherst, MA 01003 USA; 6 Pritzker School of Molecular Engineering, University of Chicago, Chicago, IL 60637 USA; 7 Universität Innsbruck, Institut für Theoretische Physik, Technikerstraße 21a, 6020 Innsbruck, Austria; 8 Department of Physics, Humboldt-Universität zu Berlin, Newtonstraße 15, 12489 Berlin, Germany; 9 Center for Hybrid Quantum Networks (Hy-Q), The Niels Bohr Institute, University of Copenhagen, Blegdamsvej 17, DK-2100 Copenhagen Ø, Denmark; 10 Department of Physics, Harvard University, Cambridge, MA 02138 USA

**Keywords:** Computational science, Quantum information, Quantum physics, Optical physics, Applied optics

## Abstract

One-way quantum repeaters where loss and operational errors are counteracted by quantum error-correcting codes can ensure fast and reliable qubit transmission in quantum networks. It is crucial that the resource requirements of such repeaters, for example, the number of qubits per repeater node and the complexity of the quantum error-correcting operations are kept to a minimum to allow for near-future implementations. To this end, we propose a one-way quantum repeater that targets both the loss and operational error rates in a communication channel in a resource-efficient manner using code concatenation. Specifically, we consider a tree-cluster code as an inner loss-tolerant code concatenated with an outer 5-qubit code for protection against Pauli errors. Adopting flag-based stabilizer measurements, we show that intercontinental distances of up to 10,000 km can be bridged with a minimized resource overhead by interspersing repeater nodes that each specialize in suppressing either loss or operational errors. Our work demonstrates how tailored error-correcting codes can significantly lower the experimental requirements for long-distance quantum communication.

## Introduction

The ability to faithfully transmit quantum information over long distances opens up new opportunities for secure communication^1,2^, sensing networks^3^, and distributed quantum computing^4^. There has been impressive progress towards the realization of quantum networks with the demonstration of long-distance entanglement distribution through satellites^5^, memory-enhanced quantum communication^6^, and a multi-node quantum network^7^. Quantum communication over intercontinental distances, however, remains a formidable challenge due to attenuation and degradation of the quantum signal as a result of loss and operational errors.

There are, in general, two classes of quantum-repeater architectures that have been proposed to overcome these obstacles^8,9^. Two-way repeaters divide the total distances into smaller links where heralded entanglement can be created in a probabilistic manner by direct photon transmission and success is heralded by two-way communication between the repeater nodes^10–12^. Such architectures require long-lived multi-mode quantum memories to reach high communication rates^13^. An alternative approach, which is the focus of this work, is to encode the quantum information in quantum error-correcting codes to battle both loss and operational (Pauli) errors as the signal is being transmitted from one repeater node to the next in a one-way architecture^14–19^. Such fault-tolerant one-way repeaters allow bridging arbitrary distances with high communication rates since the repetition rate is set by the local processing time of the repeater nodes. However, they often have daunting requirements in terms of resources needed for the physical implementation of the repeater^15,16,20,21^. Focusing on the dominant error of photon loss and abandoning fault-tolerance for operational errors can significantly relax these requirements as recently demonstrated in ref. ^17^. In particular, it was shown that by using only loss-tolerant photonic tree-cluster encoding, the bottleneck of transmission loss could effectively be overcome with only three spin qubits per repeater node. That approach is, however, not fault-tolerant against operational errors, and as a result the operational error experienced by the qubit in transmission must be very low ( ~ 10^−5^) in order to bridge intercontinental distances.

Alternatively, the concatenation of two different quantum error-correction codes has been considered to ensure fault-tolerant operation and efficiently address both loss and operational errors in quantum repeaters. In particular, the concatenation of a continuous-variable (CV) Gottesman-Kitaev-Preskill (GKP) code with small discrete-variable (DV) codes has recently been proposed^18,19,22–24^, and was shown to have increased communication performance while reducing the resource cost. While the experimental generation of optical GKP states has recently been demonstrated^25^, the construction of such states with sufficiently high quality remains a daunting challenge due to the requirements for the efficiency and performance of the hardware.

**Fig. 1:**
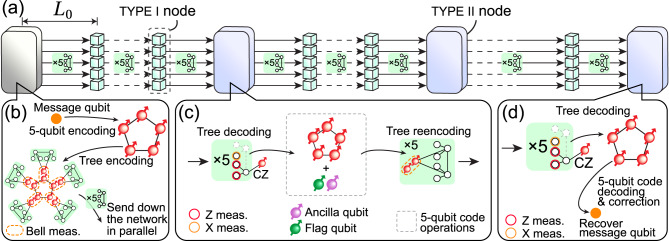
Quantum-repeater network overview. **a** Overview of the hybrid one-way quantum-repeater network containing two types of repeater nodes: TYPE I and TYPE II. **b** In the start node (grayed because no error correction is involved), a message qubit is encoded using the 5-qubit code into five data spin qubits. Then, each of those data spin qubits is encoded in parallel into a photonic tree-cluster state via a Bell state measurement (orange dashed box) with the root spin-qubit of the tree. The [2, 2] tree is used as an example here. The trees (each encased in a green box) are then sent in parallel along the repeater network where the nodes are a distance *L*_0_ apart from each other. TYPE I nodes consist of five processing blocks. Each of these blocks decodes the received tree and re-encodes the decoded qubit into a fresh tree via heralded storage^17^. **c** TYPE II nodes decode the incoming tree at the tree level, then perform stabilizer operations (i.e., two-qubit gates and syndrome extraction on the 5-qubit code level) between the decoded qubit and the ancilla qubits with accompanying flag qubits in the nodes. After the stabilizer operations, the decoded qubit is re-encoded back into a new tree and sent off to the next node. **d** At the end node, the incoming five trees are received and decoded in parallel. The 5-qubit code corrections are applied according to the syndromes obtained along the network and finally, they are decoded back into the message qubit.

In this article, we propose a purely DV-based one-way quantum-repeater architecture (see Fig. 1) that uses code concatenation and flag-based quantum error correction to achieve fault-tolerant operation in a resource-efficient manner. Specifically, we combine a loss-tolerant tree-cluster state as an inner code^17^ with a 5-qubit code operated with a flag qubit as an outer code. The generation of large photonic cluster states can be done efficiently with single emitters^26^ and has been demonstrated with a number of experimental platforms^27–30^. The 5-qubit code^31^ ensures efficient correction of operational errors for which the tree code does not provide protection. By adopting flag-based stabilizer measurements, we show that communication rates in the kHz range can be achieved over thousands of kilometers for qubit transmission errors ~10^−3^ with a minimized qubit overhead per repeater node.

Our design intersperses two types of repeater nodes that operate differently on the two codes similar to the CV-DV architecture of ref. ^18^. TYPE I nodes perform error correction solely on the tree code while TYPE II nodes perform error correction on both codes. Consequently, TYPE II nodes are more complex than TYPE I nodes. We show how the extra cost of TYPE II nodes can be included in the design of the repeater architecture to maximize network performance while minimizing the cost. Finally, we outline how both types of repeater nodes can be constructed from 5 modular processors containing only 1 quantum emitter and at most 4 memory spins each. This makes our design suitable for implementation with current quantum-network hardware such as solid-state defect centers coupled to a small qubit register of nuclear spins^32,33^.

The paper is structured as follows. In the sections “Quantum-repeater protocol” to “Decoding”, we introduce the high-level repeater protocol and its building blocks. In the section “Implementation”, we discuss a possible implementation with efficient spin-photon interfaces based on cavity-coupled quantum emitters and in the section “Repeater performance”, we quantify the performance of the repeater, which we optimize for various distances.

## Results

### Quantum-repeater protocol

The repeater architecture incorporates three main steps. First, a message qubit is encoded in an error-correction code at the starting node. This is followed by re-encoding and error correction at subsequent repeater nodes before final decoding at the end node. A high-level schematic of the entire repeater network is shown in Fig. 1, and we will now address each of the three steps separately in more detail below.

### Encoding

The message qubit at the start node is first encoded into the ⟦5, 1, 3⟧ code, otherwise known as the 5-qubit code (see Fig. 1b). Here, ⟦*n*, *k*, *d*⟧ refers to a code that encodes *k* logical qubits using *n* physical qubits with a code distance *d*. We have chosen the 5-qubit code as the outer code because it is the smallest quantum error-correcting code that can correct single arbitrary Pauli errors^31^, an attractive trait in minimizing physical resource requirements. We note that the encoding of the logical state can be performed fault-tolerantly using a scheme recently demonstrated with a diamond Nitrogen-Vacancy platform in ref. ^34^. This involves heralding the desired encoded logical state via repeated stabilizer measurements in conjunction with a flag qubit.

**Fig. 2:**
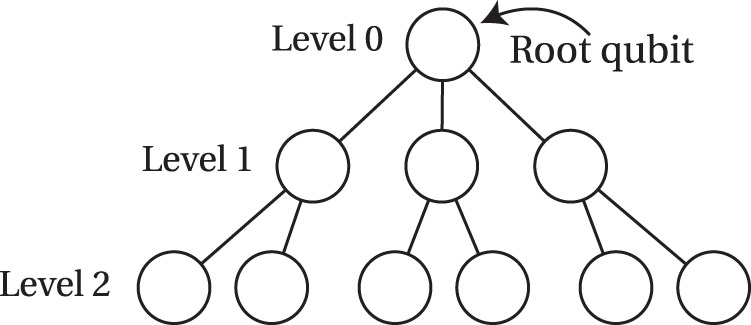
Tree-cluster state. A [3, 2] tree-cluster state is shown as an example to illustrate the different levels of a tree. Each vertex/circle represents a qubit initially prepared in the state $\left\vert +\right\rangle =(\left\vert 0\right\rangle +\left\vert 1\right\rangle )/\sqrt{2}$. The edges connecting the vertices correspond to a CPHASE gate being applied between the two qubits.

After encoding the message qubit into the outer 5-qubit code, each of the five data qubits of the code is then further encoded into a photonic tree-cluster state (inner code) that provides loss tolerance via information redundancy^35^. The tree-cluster code can be described by its branching vector **t** = [*b*_0_, *b*_1_, …, *b*_*d*_] which determines the degree of branching from each level in the tree beginning from the root qubit (see Fig. 2). Every node in the tree-cluster state described by the branching vector represents a qubit in state $\left\vert +\right\rangle =(\left\vert 0\right\rangle +\left\vert 1\right\rangle )/\sqrt{2}$ and each of the edges represents CPHASE gate. This resulting tree-cluster state is then the unique state that is stabilized, i.e., it has eigenvalue + 1 for each of the operators ${K}_{\nu }={X}_{\nu }{\prod }_{b\in {{{{\mathscr{N}}}}}_{\nu }}{Z}_{b}$, where *ν* labels the qubits of the tree state and ${{{{\mathscr{N}}}}}_{\nu }$ denotes the set of qubits connected to the *ν*^th^ qubit. To encode a data qubit in the tree-cluster code, a single Bell state measurement of the root qubit of the tree-cluster state and the respective data qubit is sufficient as detailed in ref. ^17^ and shown in Fig. 1b.

At this point, the message qubit has been encoded into 5 photonic tree-cluster states, which are sent down the repeater network in parallel (see Fig. 1a). Note that we are assuming a generation scheme of the photonic tree-cluster states where each photon of the tree is generated sequentially from a single emitter featuring a multi-level electronic ground state manifold^26^, hence the photons are sent down the optical fibers using time-bin encoding. For details on this scheme, we refer to ref. ^17^. Consequently, the 5 trees can be transmitted using 5 single-mode fibers.

### Re-encoding and error correction

In the repeater network, the incoming photonic qubits from the previous node will be received by either a TYPE I or TYPE II repeater node, each specializing in dealing with types of errors. The TYPE I nodes only operate on the inner tree code to correct transmission loss, and we envision that each TYPE I node consists of 5 parallel repeater subnodes similar to the nodes considered in ref. ^17^. Each of these 5 subnodes receives and operates on 1 of the 5 tree-encoded qubits of the outer 5-qubit code. Their basic operation is to decode each incoming photonic tree-encoded data qubit into a spin-qubit and then re-encode it into a new photonic tree encoding. In this process, loss will be corrected but not logical errors on the outer 5-qubit code level.

The re-encoding of the data qubit into the new tree-cluster state is achieved with a Bell state measurement between any one of the first-level photonic qubits in the incoming tree and the root qubit of the new tree along with the measurement of the remaining qubits in the incoming tree in appropriate single-qubit bases according to the stabilizer generators of the tree (see section “Encoding”). The whole procedure can be performed in a loss-tolerant way with only two memory spins and a single cavity-coupled emitter as outlined in ref. ^17^. Note that we assume that decoherence errors in all memory spins considered in this article are negligible compared to other gate errors because the information is only stored short-term in the memory spins in our network protocol.

Importantly, the Bell state measurement between a first-level photonic qubit of the incoming tree and the stationary root qubit of the new tree is not protected against faulty gate operations, which introduces re-encoding error *ϵ*_r_ on our encoded qubit on the tree-code level. In addition, we assume that each qubit in the trees is subjected to a single-qubit depolarizing error of *ϵ*_0_, which comes from the inherent operational error in the underlying hardware. The presence of this re-encoding error is why the scheme in ref. ^17^ is not fault-tolerant and requires very low re-encoding error rates for long-distance communication. Here, we solve this issue with the outer 5-qubit code, which provides an extra layer of protection for the message qubit in the TYPE II repeater nodes.

The TYPE II repeater nodes are designed to primarily correct for the accumulated re-encoding errors in the network by operating on both the inner tree encoding and the outer 5-qubit encoding. We refer to the accumulated re-encoding error between TYPE II nodes as the transmission error *ϵ*_trans_. This transmission error experienced by the encoded qubit is modeled via the single-qubit depolarizing channel discussed in Methods “Error model”. The first step to correct for *ϵ*_trans_ is to decode the tree encoding using the same procedure as the TYPE I nodes thereby correcting loss errors. Once the 5 incoming trees are decoded into 5 single spin qubits, the syndrome extraction of the 5-qubit code is performed in a fault-tolerant manner using a flag qubit to correct for *ϵ*_trans_. For the fault-tolerant error-correction protocol, refer to Methods “Fault-tolerance”. After the syndrome extraction, the decoded qubits are re-encoded back into newly generated trees and are sent down the network. Note that since a TYPE II node also performs the same decoding and encoding procedure as a TYPE I node, we also take into account the errors generated by these steps in the transmission error. Refer to Methods “Error model” for how these are taken into account.

Additionally, we consider noisy two-qubit gates in TYPE II nodes, which we model via a two-qubit depolarizing channel with an error rate of *ϵ*_0_ discussed in Methods “Error model”. Note that, as we will discuss in the section “Implementation”, there are two-qubit gates in each of the TYPE II nodes that have to be performed in a teleported manner, which have an error rate of 3*ϵ*_0_ because each of them is essentially comprised of 3 two-qubit gates.

In the section “Repeater performance”, we discuss that the inherent operational error is related to the re-encoding error via the relation *ϵ*_r_ ≈ 3*ϵ*_0_ found through our numerical simulation (see Methods “Error model”). This means with the increase of the re-encoding error, the two-qubit gates in TYPE II nodes become noisier, and the placements of TYPE II nodes in the network would become more sparse. Note that we assume errors introduced by single-qubit gates are negligible since they are typically much smaller than errors induced by two-qubit gates in an architecture based on diamond defects^36^. We also assume that the dominant errors in the qubit readout operations enter through the noisy two-qubit gates because the qubit readout operations considered in this paper are always associated with stabilizer operations of the 5-qubit code. Therefore, the qubit readouts themselves are considered error-free. In summary, the faulty re-encoding procedure in both TYPE I and TYPE II nodes, and the noisy two-qubit gates in the TYPE II nodes are the only source of operational errors in our model.

Besides correcting for transmission errors, the 5-qubit code can also correct for failed decoding attempts of the photonic tree-cluster states due to the loss of too many photons. In general, any quantum error-correcting code capable of correcting *t* arbitrary Pauli errors could also be used to correct 2*t*-erasure errors^37^. Consequently, the 5-qubit code can correct for up to two failed decoding attempts (erasure errors) of the incoming photonic tree-states. While the probability of 2-erasure errors occurring within the same TYPE II node is very small due to the loss correction of the TYPE I nodes, we find that correcting for a single lost tree, i.e., 1-erasure errors, at the TYPE II nodes can significantly boost the communication rate (see Supplementary Notes 1 and 3). We, therefore, apply the erasure-error correction in this work in the section “Repeater performance”, for more details please refer to Methods “Erasure-error correction”.

### Decoding

The extracted syndromes of the error correction, which provide information about whether a 1-erasure error has occurred or not, are simply stored in a classical register. This register is classically communicated to the end node where it is interpreted and only when required single-qubit gates are applied to correct the errors according to the interpreted syndromes. Thus, it is not necessary to perform any correction on the quantum level at the repeater nodes. This allows us to avoid additional decoherence due to latency introduced by applying the error correction at every TYPE II node and allows for performing corrections based on the joint information of all error syndromes from the repeater network. This approach is known as *Pauli frame updating*^38^.

The end node is a TYPE II node, where the tree-cluster states are first decoded into the 5 data spin qubits. Error correction is then performed on the 5 data spin qubits according to the syndromes extracted throughout the network, after which they are then further decoded back into the original message qubit and measured. Note that since the encoding and decoding procedure on the 5-qubit code level is only done at the start and end node, we assume that errors introduced at these steps are negligible.

### Implementation

We will now discuss a specific modular design of the repeater nodes that allow for implementation with small qubit processors containing 1 cavity-coupled quantum emitter and at most 4 memory spins. Our choice of a modular design is motivated by current hardware capabilities with, e.g., solid-state defect centers where efficient spin-photon interfaces can be achieved through coupling to nanophotonic resonators^32,33,39^. By coupling to a few near-by nuclear spins, such small processors can be envisioned. Coupling between the processors (needed for TYPE II nodes) can be achieved through photon-mediated interactions between the emitters as we outline below.

As described above, a TYPE I node consists of 5 parallel repeater subnodes similar to the node described in ref. ^17^, with each subnode requiring only 2 memory spins and 1 quantum emitter for the generation of depth-3 photonic tree-cluster states to perform the loss-tolerant re-encoding operation.

The photonic tree generation follows the scheme of ref. ^26^, where the photons of the tree are sequentially emitted from the emitter with intermediate entangling operations between the emitter and the 2 memory spins. In this way, the branches of the tree are generated one by one starting from the bottom. We note that this generation scheme requires a subsequent re-ordering of the photons using optical switching and a delay line as detailed in ref. ^17^ such that the first-level photons lead when arriving at the next repeater node. This is necessary since the absence/presence of a first-level photon determines the measurement bases of the remaining photons of the branch.

A key element for the re-encoding operation is a cavity-mediated CPHASE gate between an incoming photon and the emitter^40–43^. The basic principle of this gate is that if only one of the ground states of the emitter is coupled to the cavity mode, an incoming photon will be reflected with/without a *π*-phase shift if the emitter is in the uncoupled/coupled state. This operation allows for transferring the quantum state of a photon to the emitter, heralded by subsequent detection of the photon. As shown in ref. ^17^, this makes the re-encoding operation robust to transmission losses by first transferring the state of a first-level photon of the incoming tree to the emitter in a heralded way followed by a Bell state measurement between the emitter and a memory spin. The memory spin constitutes the root qubit of a fresh tree-cluster state emitted prior to the reception of the incoming tree.

**Fig. 3:**
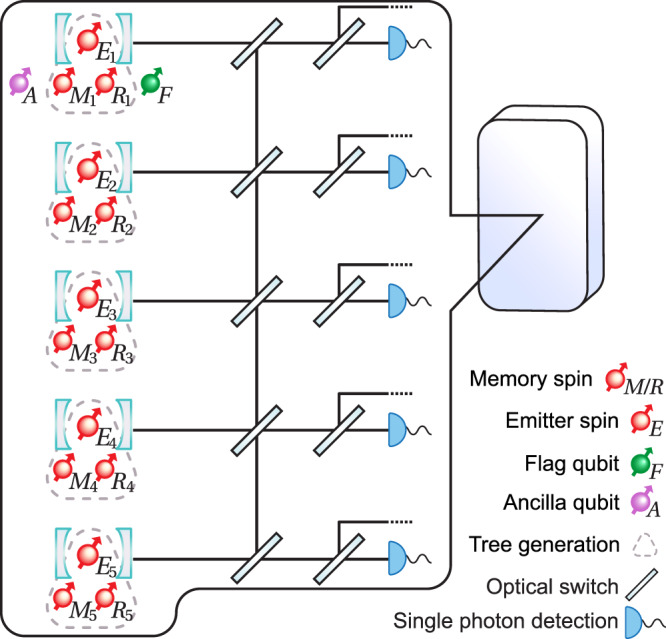
Physical resources of the TYPE II repeater nodes. 5 sets of single-sided cavities, each with a quantum emitter *E*_*j*_ with *j* ∈ {1, 2, 3, 4, 5} and accompanying memory spins. The 5 emitter spins host the decoded qubits from the 5 trees. They are equipped with optical switches that allow for performing teleported CNOT gates between the desired decoded qubits that are physically far apart. In all the sets, 2 extra qubits, i.e., *M*_*j*_ and *R*_*j*_, are needed for tree generation and teleported two-qubit gates. In one of the sets, another 2 qubits are needed for the ancilla and flag qubits, denoted by *A* and *F*, respectively.

The requirements of TYPE II nodes are different from the TYPE I nodes since they perform error correction on the outer 5-qubit code in addition to the loss correction on the inner tree-cluster code. Here we outline how a modular architecture can enable this increased functionality with a very modest increase in resources compared to TYPE I nodes. Similar to TYPE I nodes, we consider an implementation with 5 cavity-coupled emitters that each can receive and generate their tree-cluster states using 2 additional memory spins per emitter (see Fig. 3). To allow for the syndrome extraction of the 5-qubit code, we only need one of these emitters to be coupled to 2 extra memory spins, which act as ancilla and flag qubits of the 5-qubit code. Consequently, we require a sequential extraction of the syndromes, which increases the duration of the error-correction procedure. A faster parallel extraction would be possible with the added expense of more ancilla and flag qubits, but here we choose to focus on the sequential operations to minimize the number of qubits per repeater node.

**Fig. 4:**
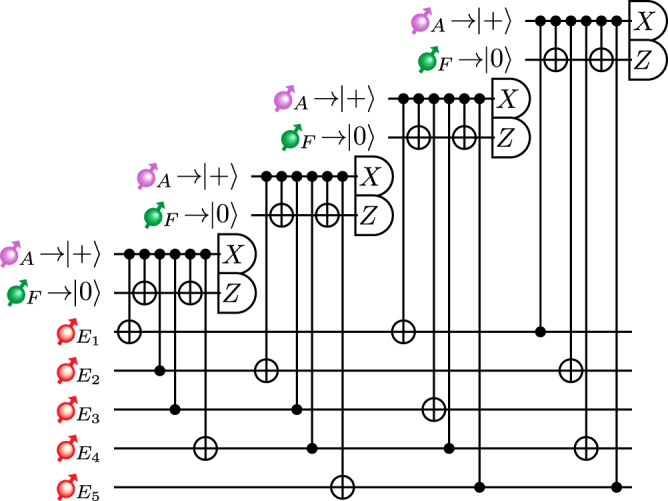
Error-correction circuit. The circuit that a TYPE II node performs to fault-tolerantly correct for operational errors using the 5-qubit code. For the fault-tolerant error-correction protocol, refer to Methods “Fault-tolerance”.

We assume direct coupling between an emitter and its near-by memory spins based, for example, on spin-spin interactions, which allows for the implementation of multi-qubit gate operations. However, as shown in Fig. 4, the syndrome extraction of the 5-qubit code requires two-qubit gates between the ancilla qubits belonging to different emitter-cavity systems and the data spin-qubit. To implement such non-local operations, teleported gates can be used.

**Fig. 5:**
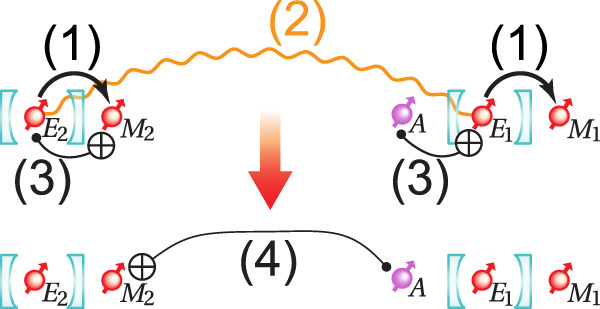
Teleported CNOT gate. Procedure for performing a teleported CNOT gate between an ancilla qubit (control) and a data qubit (target) by heralding a Bell pair between two emitters and performing a measurement on the Bell pair. For simplicity, not all spins coupled to each cavity are shown (Fig. 3). We use the realization of a teleported CNOT gate between an ancilla qubit *A* and the decoded qubit in *E*_2_ as an example. The steps are as follows: (1) The decoded qubits in the emitter spins *E*_*j*_ are transferred to the auxiliary memory spins *M*_*j*_. (2) A Bell pair is heralded between the two emitter spins. (3) Local spin-spin CNOT gates are performed between spins of interest. (4) The qubits corresponding to the Bell pair heralded in step (2) are measured to perform a teleported CNOT gate between desired control and target qubits up to some Pauli gate(s) correction according to the measurement outcome.

The teleported CNOT gate involves heralding a Bell pair between two emitters via the transmission and subsequent reflection of a photon^44–47^ (see Fig. 5). The creation of such a Bell pair can be performed using the same operations and same spins, which are shown enclosed in dashed gray triangles in Fig. 3, to decode an incoming tree-cluster and generate a new tree-cluster. Note that we can only perform the Bell pair creation once the fresh tree has been generated with the memory spin *R* hosting the root qubit and the emitter spin is “freed”, i.e., reinitialized. Specifically, for creating a Bell pair, a photon entangled with one of the emitters is generated by emission and then scattered off another cavity-spin system followed by a heralding measurement of the photon. This will prepare the two emitter spins in a Bell state, which can be used to mediate a gate between two remote spin qubits through gate teleportation. To perform teleported CNOT gates between two qubits of interest, local CNOT gates between the qubits of interest and the emitter spins are performed followed by single-qubit measurements of the emitter spins. Note that the emitter spins in the repeater nodes hold the decoded data qubits upon the reception of trees, therefore we need to first free them up by transferring the decoded data qubits onto the auxiliary memory spins as shown in Fig. 5 before performing the CNOT gate. This will then amount to a CNOT operation between the two distant spin qubits up to a single-qubit correction dictated by the measurement outcome. In order to connect the pairs of cavity-emitter systems dictated by the error-syndrome-extraction circuit (see Fig. 4), we imagine that fast optical switching^48^ is employed to ensure the generation of Bell pairs between the respective emitter spins.

### Repeater performance

In this section, we benchmark the performance of the repeater network by looking at the secret key rates that are achieved when executing a Quantum Key Distribution (QKD) protocol. This quantity encapsulates both the fidelity of the final message qubit and the raw bit rate of the network. Therefore, the determination of the secret key rate provides an excellent means of assessing the general performance of the network. Since our repeater network is based on the one-way quantum-repeater protocol, we consider the prepare-and-measure-based six-state protocol^49^ as the most suitable QKD protocol, whose secret key fraction is dependent on the effective error rate of the message qubit at the end node. Details about this secret key fraction are given in Methods “Repeater performance”.

Furthermore, we boost the secret key rate by leveraging the 5-qubit code’s ability to correct for erasure errors as explained in the section “Re-encoding and error correction”. Note that our secret key rate depends on 2 facts: (1) the effective error rate at the end node depends on how many erasure errors occurred in the network, and (2) the presence of erasure errors results in a different successful transmission probability of the message qubit through the network (see Methods “Repeater performance”). Recall that we only consider correcting for 1-erasure errors and not 2-erasure errors at the 5-qubit code level for reasons explained in the section “Re-encoding and error correction”. These dependencies are accounted for in the secret key rate calculation by performing a weighted sum over the possible number of 1-erasure-error occurrences in the network, effectively resulting in an average secret key rate

1 $$\,{{\mbox{SKR}}}\,={\tau }_{{{{\rm{tot}}}}}^{-1}\mathop{\sum }\limits_{i=0}^{{m}_{{{{\rm{II}}}}}}\left(\begin{array}{c}{m}_{{{{\rm{II}}}}}\\ i\end{array}\right){f}_{{m}_{{{{\rm{II}}}}},i}\,{p}_{{{{\rm{trans}}}}}({m}_{{{{\rm{II}}}}},i),$$

where *m*_I_ (*m*_II_) is the number of TYPE I (TYPE II) nodes, ${f}_{{m}_{{{{\rm{II}}}}},i}$ is the secret key fraction, *p*_trans_(*m*_II_, *i*) is the probability of successful transmission with 1-erasure errors in *i* distinct TYPE II nodes (see Methods “Repeater performance”), and

2 $${\tau }_{{{{\rm{tot}}}}}={\tau }_{{{{\rm{tree}}}}}+14{\tau }_{{{{\rm{ss}}}}}+26{\tau }_{{{{\rm{tele}}}}}+8{\tau }_{{{{\rm{meas}}}}},$$

is the total processing time of each TYPE II node for 1 logical qubit. Here, *τ*_ss_ is the local spin-spin two-qubit gate time, *τ*_tele_ is the teleported two-qubit gate time, and *τ*_meas_ is the spin readout time. We arrived at eq. (2) by considering the longest possible time taken by the fault-tolerant error-correction protocol since that will become the processing bottleneck of the network (see Supplementary Note 4). We assume that the readout of the ancilla and flag qubit can be done simultaneously. Finally, a new tree-cluster has to be generated in the TYPE II node with tree vector **t** = [*b*_0_, *b*_1_, …, *b*_*d*_] which takes time *τ*_tree_ and is estimated as^17^

3 $$\begin{array}{l}{\tau }_{{{{\rm{tree}}}}}\,\approx \,{b}_{0}\left[100+{b}_{1}(1+{b}_{2}(1+\cdots {b}_{d-1}(1+{b}_{d})\cdots \,))\right]{\tau }_{{{{\rm{ph}}}}}\\ \qquad\quad +{b}_{0}\left[3+{b}_{1}(1+{b}_{2}(1+\cdots {b}_{d-2}(1+{b}_{d-1})\cdots \,))\right]{\tau }_{{{{\rm{ss}}}}},\end{array}$$

where *τ*_ph_ is the emission time of a single photonic qubit and the emission time of the first-level photons is assumed to be 100*τ*_ph_. The longer emission time of the photons means a narrower frequency bandwidth. This is necessary since the first-level photons may participate in a cavity-mediated CPHASE gate at the next repeater station. If the frequency bandwidth of the incoming photon is not narrow compared to the cavity (Purcell) enhanced linewidth of the emitter, the gate operation will be imperfect. Assuming an emission time of 100*τ*_ph_ will ensure that these imperfections lead to gate errors ≲ 10^−4^ ^17^.

In principle, TYPE I nodes could operate faster than TYPE II nodes since they are only performing loss correction at the tree-code level. However, TYPE I nodes would have to wait for the TYPE II nodes to complete their operations before transmitting the tree-encoded qubits. Thus, the operation time of the TYPE II nodes becomes the bottleneck that sets the repetition rate of the repeater network and faster TYPE II nodes are not required. As a consequence, the hardware architecture at TYPE I and II nodes can be quite similar, facilitating the large-scale building of such nodes.

To find the optimal configuration of the repeater network, that is to maximize the secret key rate while using as few physical resources as possible, we perform a numerical minimization of a dimensionless cost function for a specific total distance

4 $$C={{{\mbox{SKR}}}}^{-1}\frac{{L}_{{{{\rm{att}}}}}}{{\tau }_{{{{\rm{ph}}}}}{L}_{{{{\rm{tot}}}}}}({m}_{{{{\rm{I}}}}}+\kappa {m}_{{{{\rm{II}}}}}),$$

over the parameters *L*_0_, *m*_II_, and **t** with constraints discussed in Methods “Numerical minimization”. Note that for simplicity, we assume a uniform interspersing of TYPE I and TYPE II nodes in the repeater network. For more details on how the repeaters are interspersed, refer to Supplementary Note 8. The coefficient *κ* is the relative cost of a TYPE II to a TYPE I node. In a repeater network with multiple types of nodes, one type of node may require more functionality (as is the case here) and thus be more expensive than another. For instance, if *κ* = 1, then the cost of a TYPE I node is the same as that of a TYPE II node.

To quantify the cost per unit length, we divide the number of nodes in the cost function with the total distance between the start and end node *L*_tot_. This is expressed in units of the attenuation length *L*_att_ to make the cost function dimensionless. We also quantify the cost per unit of time by including the inverse of the secret key rate (SKR) which is in unit Hertz. Note that the secret key rate depends on the photon emission time *τ*_ph_, therefore its presence in the denominator in eq. (4) serves to make the cost function dimensionless. It also means that we are expressing the cost per unit time in units of the photon emission time. The values for the constants used are shown in Table 1.

**Table 1:** Repeater specifications.

Quantity			Value
Spin-spin gate time,	*τ*_ss_	♢	100 ns
Single photon emission time,	*τ*_ph_	♢	1 ns
Spin readout time,	*τ*_meas_		1 μs
Teleported two-qubit gate time,	*τ*_tele_		1 μs
Optical fiber’s attenuation length,	*L*_att_	♢	20 km
Effective photon-detection efficiency,	*η*_d_	♢	0.95

Values of the quantities used in the minimization of the cost function are shown in eq. (4). Note that we assume the availability of efficient frequency conversion to the telecom band such that the attenuation length in optical fibers is 20 km. The symbol ♢ denotes that the values were also used in ref. ^17^. The effective photon-detection efficiency, *η*_d_ is the combined efficiency of in/out-coupling of the photon, frequency conversion, and the efficiency of the photon detectors.

**Fig. 6:**
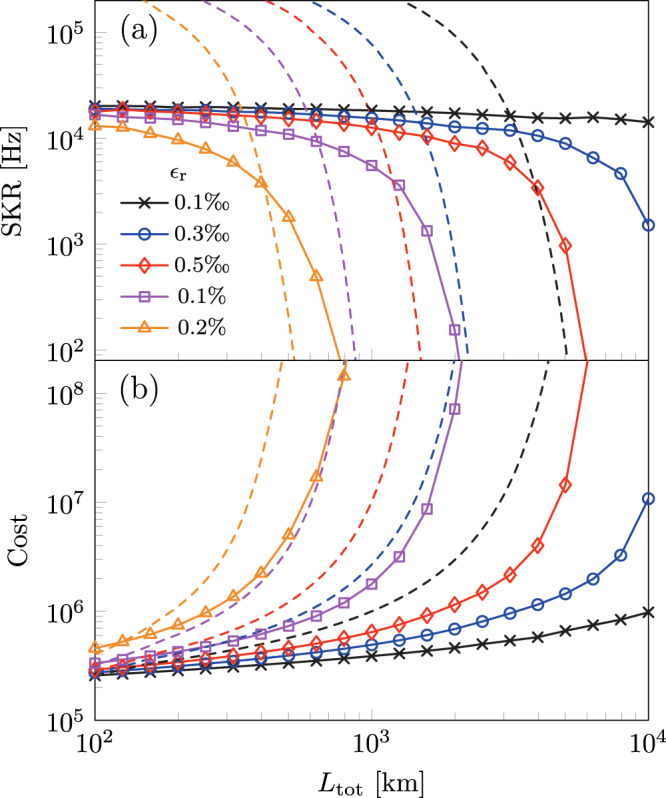
Repeater network performance. **a** The secret key rate SKR corresponding to **b** the minimized cost function ${C}_{\min }$ as a function of the distance *L*_tot_ for various re-encoding error probabilities *ϵ*_r_ with fixed relative node weight *κ* = 1. For comparison, the secret key rates and the costs of the homogeneous repeater network from ref. ^17^ multiplied by a factor of 5 are included as dashed lines as well. The factor of 5 is included to compensate for the fact that the concatenated repeater is similar to 5 parallel homogeneous repeater networks. The cost function of the homogeneous repeater is the same as eq. (4) with *m*_II_ = 0, and was minimized independently.

The results of the optimizations for different error rates are shown in Fig. 6 for *κ* = 1. For short distances, the homogeneous repeater scheme from ref. ^17^ (dashed lines) is superior since it does not possess the time overhead of error correction that comes with TYPE II nodes and thus enables a higher repetition rate. For longer total distances, however, the secret key rate of the concatenated repeater protocol (solid lines with markers) greatly surpasses that of the homogeneous repeater protocol of ref. ^17^ due to the added protection from the outer 5-qubit code. For instance, for *ϵ*_r_ = 0.1% (purple line), the secret key rate for the concatenated repeater is ~5.5 kHz at 10^3^ km, while for the homogeneous counterpart (dashed line), the rate at the same total distance is less than 1 Hz. Furthermore, despite the higher secret key rates of the homogeneous repeater scheme, we see that from Fig. 6b its cost is significantly higher than that of the concatenated repeater protocol. This means that the secret key rate itself is not the only relevant indicator of overall performance, but the cost associated with building the proposed network needs to be considered as well. Note that even if the cost function is ignored and if the repeater network is configured by purely maximizing the secret key rate, the gain in the secret key rate would be minimal with the tradeoff being a significantly increased cost (see Supplementary Note 5).

**Fig. 7:**
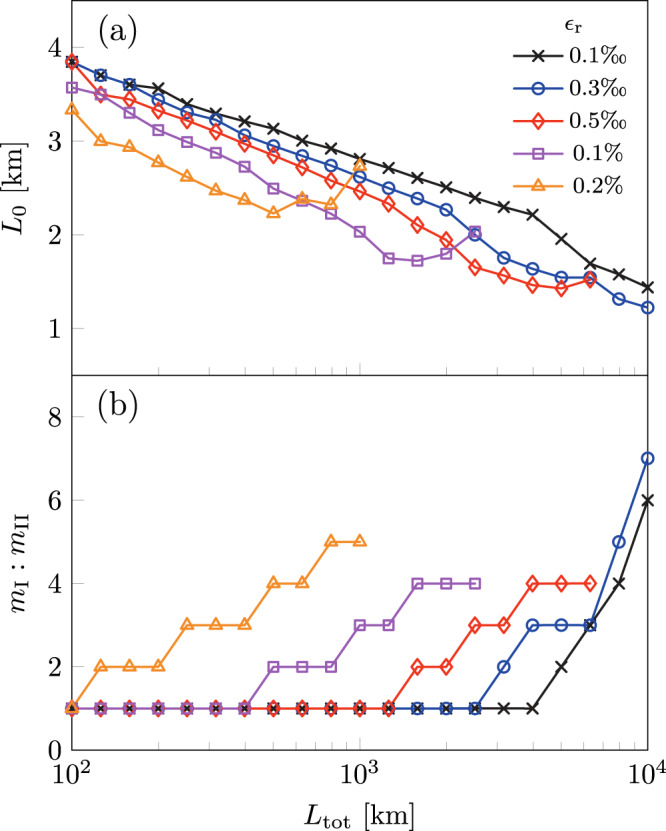
Repeater ratio and spacing. **a** The inter-repeater distance *L*_0_, and **b** the ratio of the number of TYPE I to TYPE II nodes *m*_I_: *m*_II_ as a function of total distance corresponding to Fig. 6.

The accompanying results in Fig. 7a reveal the optimal inter-repeater distances required to achieve the best secret key rates whilst maintaining minimum cost. When considering higher re-encoding error probabilities *ϵ*_r_ for fixed total distances, the optimum inter-repeater distance *L*_0_ decreases to minimize the loss error to counter the increasing re-encoding error rate. From Fig. 7b, we see that the configuration with the highest re-encoding error has the lowest proportion of TYPE II nodes in the network, even though it has the lowest inter-repeater distance. This is because the re-encoding error rate scales with the error rate of the two-qubit gates in the TYPE II nodes as explained in the section “Re-encoding and error correction”. Therefore, adding more TYPE II nodes no longer entails better error suppression, but instead introduces more error in the network. Hence, we find an asymmetry between the number of TYPE I and TYPE II nodes as the ratio of *m*_I_ to *m*_II_ deviates from unity. This result demonstrates that even when we consider the cost of TYPE I and TYPE II nodes to be equal, i.e., *κ* = 1, the asymmetry between loss and operation errors in the repeater network is still present. Note that it is in principle possible for the ratio of *m*_I_ to *m*_II_ to have values below unity. In our calculation, however, because of the constraint on the *m*_II_ set, the minimum value that this ratio can attain is unity.

**Fig. 8:**
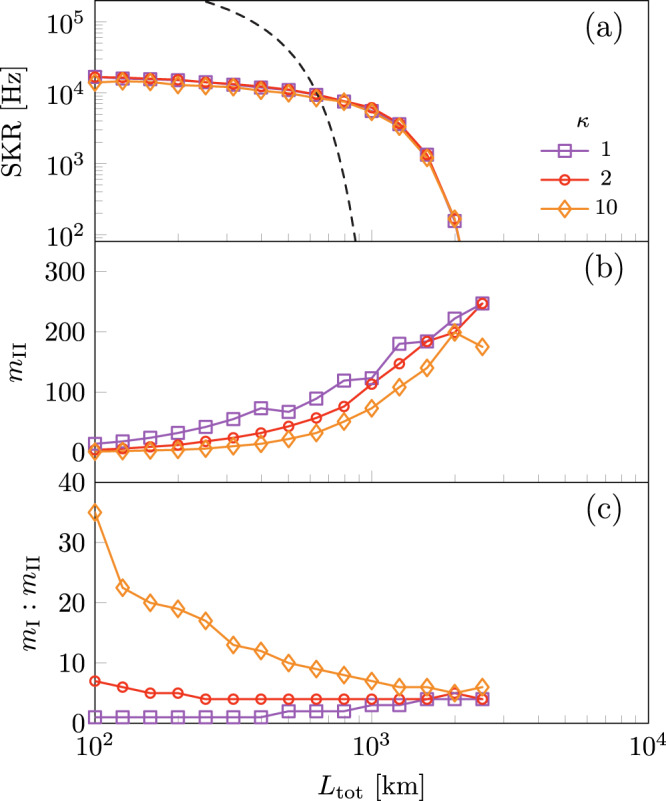
Repeater weight characterization. **a** The secret key rate SKR corresponding to the minimized cost function ${C}_{\min }$ as a function of the distance *L*_tot_ for various relative node weights *κ* with fixed re-encoding error probability *ϵ*_r_ = 0.1%. For comparison, the secret key rate of the homogeneous repeater network multiplied by a factor of 5 from ref. ^17^ is included as a dashed line optimized with respect to the cost function shown in eq. (4) with fixed *m*_II_ = 0. **b** The corresponding number of TYPE II nodes in the network and **c** the corresponding ratio of the number of TYPE I to TYPE II nodes as a function of the total distance.

To demonstrate how the value of *κ* > 1 influences the cost and secret key rate when considering TYPE II nodes that are more expensive than TYPE I nodes, we optimize with *κ* ∈ {1, 2, 10} for fixed re-encoding error rate *ϵ*_r_ = 0.1% in Fig. 8. As the value of *κ* increases, we see that the secret key rate is only minimally affected as shown in Fig. 8a whilst the number of TYPE II nodes in the network is significantly decreased as shown in Fig. 8b. From Fig. 8c, we infer that this is followed by an increase in the number of TYPE I nodes in the network. This is expected as TYPE II nodes are more costly. Additionally, Fig. 8c shows how the ratio of TYPE I to TYPE II nodes changes as a function of the distance. For high-cost TYPE II nodes (*κ* = 10) the ratio decreases with distance. This can be understood from the accumulation of re-encoding errors in the network. For small distances, the accumulated error is relatively small, and it is therefore advantageous to employ a few TYPE II nodes. For larger distances, the accumulated error necessitates more TYPE II nodes despite their higher cost. On the other hand, we observe that the ratio of TYPE I to TYPE II nodes increases with distance even for a low cost of TYPE II nodes (*κ* = 1). We attribute this to the fact that placing too many TYPE II nodes in the network introduces more operational errors than it can suppress.

## Discussion

In conclusion, we have outlined how a resource-efficient DV one-way quantum repeater can be constructed by concatenating a flag-based 5-qubit code with a loss-tolerant tree-cluster code. The high loss tolerance and conceptually straightforward encoding/decoding of the tree-cluster code make it suitable as an inner code that protects against photon loss in transmission between repeater nodes. An extra layer of protection is provided by the outer 5-qubit code which effectively suppresses operational errors that accumulate due to the non-fault-tolerant nature of the tree-cluster code against Pauli errors. As a result, the code-concatenated repeater is able to bridge distances of several thousand kilometers for re-encoding error probabilities as high as ~ 0.1%.

The code concatenation allows for an architecture tailored to the asymmetry between loss and operational errors that are characteristic of quantum communication. Specifically, we have shown how the optimizations can include the relative cost of the different repeater nodes to arrive at an optimized architecture with many relatively cheap TYPE I nodes that only correct errors due to transmission loss, but far fewer expensive TYPE II nodes that also correct operational errors. In particular, we found that such an optimization could be done with a minimal effect on the optimal secret key rate of the repeater network.

This represents a major advantage because it achieves fault-tolerant operation with a modest resource overhead and allows for long-distance communication with error rates roughly an order of magnitude larger compared to the non-fault-tolerant scheme of ref. ^17^. Furthermore, the DV nature of our protocol circumvents the highly challenging generation of optical GKP states, making our proposal applicable to a wide range of experimental systems, which are predominantly qubit-based. In Supplementary Note 11, we provide further details on the comparison between our work and previous repeater protocols outlining how our scheme has a minimized number of spin qubits per repeater node. Due to the flexibility of our architecture, it is possible to use other quantum error-correcting codes such as the ⟦7, 1, 3⟧ and ⟦9, 1, 3⟧ codes or the ⟦4, 2, 2⟧ quantum error detecting code with minimal modification. We leave it up to future work to investigate if such approaches could lead to better results in terms of the choice of code, network performance, and resource requirement.

We have outlined a possible modular implementation of the repeater based on few-qubit processors with an efficient spin-photon interface through a cavity-coupled quantum emitter. Solid-state systems such as group-IV defect centers in diamond^6,33,50^ are promising hardware candidates having demonstrated both efficient coupling to nanophotonic cavities^6^ and access to small-scale qubit registers through coupling to near-by nuclear spins^33^. To implement both TYPE I and TYPE II nodes, only 5 cavity-emitter systems are required. Importantly, it is sufficient for each of these systems to have just 1 cavity-coupled emitter spin with direct, e.g., magnetic, coupling to 4 near-by nuclear spins. Operations between the processors, which are needed for the TYPE II nodes can be achieved through teleported gates facilitated by photon-mediated interactions between the quantum-emitter spins.

## Methods

### Fault-tolerance

**Fig. 9:**
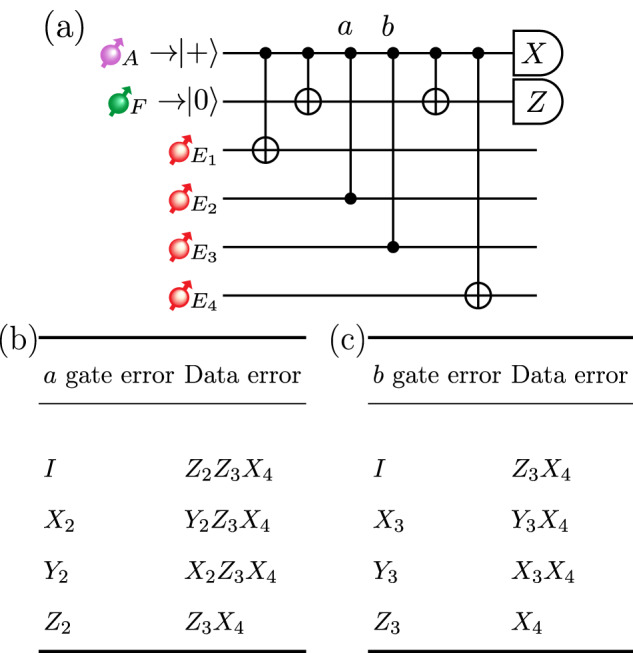
Error propagation in the circuit. **a** Circuit diagram of the error propagation in a subsection of the 5-qubit code circuit. **b** The errors induced by gate *a* if it was triggered by an error on the ancilla qubit and **c** similarly for gate *b*. Example taken from ref. ^51^.

In a TYPE II node, an additional flag qubit in a TYPE II node is used to perform error correction, with a circuit shown in Fig. 4. This additional qubit aids in suppressing the otherwise undetectable physical errors propagated by faulty gates in the stabilizer operations^51^. In Fig. 9, we consider a subsystem of Fig. 4 as an example and illustrate how an error that occurred right before a two-qubit gate during the stabilizer operation could result in logical errors on the data qubits due to the error being propagated by the two-qubit gate onto the data qubits. Then, we consider additional errors induced by the noisy two-qubit gate on the target qubit. With one additional flag qubit prepared in state $\left\vert 0\right\rangle$, the propagated error on the data qubits would then be detected because the flag qubit would be measured as being in state $\left\vert 1\right\rangle$. When the flag qubit is detected as being in state $\left\vert 1\right\rangle$, we switch over to the unflagged circuit and apply the Pauli operations according to the syndromes measured during this unflagged circuit to revert the propagated error. An example is shown in Fig. 9b, c. For the complete fault-tolerant error-correction protocol, see the Supplementary Note 2.

### Erasure-error correction

In the event of a 1-erasure error being heralded at a TYPE II node, we initialize the spin in the node corresponding to the lost tree to state $\left\vert 0\right\rangle$, while the rest of the intact trees are decoded into spins in the other nodes as usual. The 5-qubit code stabilizer operations are then performed on the 5 qubits, projecting the mixed state back into the logical codespace of the 5-qubit code, up to some Pauli corrections on the 5 qubits. For example, if the 5^th^ qubit that was part of the encoded 5-qubit logical state $\left\vert {\psi }_{L}\right\rangle$ was lost, then the original encoded state would be restored according to the Pauli corrections in Table 2. The procedure is similar for correcting a 2-erasure error, however, we have not included corrections of 2-erasure errors in our optimizations since they occur very rarely compared to 1-erasure errors.

**Table 2:** Effect of erasure error on projected state.

Syndrome	Projected state
$\left\vert ++++\right\rangle$	$\left\vert {\psi }_{L}\right\rangle$
$\left\vert ++--\right\rangle$	${X}_{5}\left\vert {\psi }_{L}\right\rangle$
$\left\vert +-++\right\rangle$	${Z}_{5}\left\vert {\psi }_{L}\right\rangle$
$\left\vert +---\right\rangle$	${X}_{5}{Z}_{5}\left\vert {\psi }_{L}\right\rangle$

The syndrome observed from reading out the ancilla qubits and the corresponding restored encoded logical states assuming the 5th qubit was lost.

To complete the error correction look-up table, we considered the cases in which additional Pauli errors also occurred on other non-lost qubits. The resulting look-up table is shown in Table 3. Note that this look-up table is not unique, i.e., multiple distinct errors could lead to the same syndrome. For instance, if the 1st qubit is lost, the syndrome $\left\vert -+++\right\rangle$ corresponds to the cases where either an *X*_2_, *Z*_3_, *Z*_4_, or *X*_5_ error also occurred. Since they are indistinguishable due to the shared syndrome, we choose to only correct for *X*_2_. Randomly choosing one out of the four possible errors to correct does not affect the suppression of error because we are considering the depolarizing noise model where every Pauli error occurs with the same probability. However, if one is considering a biased noise model, it is wise to choose which case to correct in order to maximize the error suppression.

**Table 3:** Erasure-error correction look-up table.

	Correction			
Syndrome	Lost qubit:	1^st^/2^nd^	3^rd^/4^th^	5^th^
$\left\vert ++++\right\rangle$		*I*	*I*	*I*
$\left\vert +++-\right\rangle$		*X*_1_	*Y*_3_*Y*_4_	*X*_1_
$\left\vert ++-+\right\rangle$		*Z*_1_*X*_2_	*Z*_3_	*X*_1_*X*_5_
$\left\vert ++--\right\rangle$		*Y*_1_*X*_2_	*X*_3_*Y*_4_	*X*_5_
$\left\vert +-++\right\rangle$		*X*_1_*Z*_2_	*Z*_3_*X*_4_	*Z*_5_
$\left\vert +-+-\right\rangle$		*Z*_2_	*X*_3_*Z*_4_	*Z*_1_*X*_5_
$\left\vert +--+\right\rangle$		*Y*_1_*Y*_2_	*X*_4_	*X*_1_*Y*_5_
$\left\vert +---\right\rangle$		*Z*_1_*Y*_2_	*Y*_3_*Z*_4_	*Y*_5_
$\left\vert -+++\right\rangle$		*X*_2_	*Y*_3_*X*_4_	*Y*_1_*X*_5_
$\left\vert -++-\right\rangle$		*X*_1_*X*_2_	*Z*_4_	*Z*_1_*X*_5_
$\left\vert -+-+\right\rangle$		*Z*_1_	*X*_3_*X*_4_	*Z*_1_
$\left\vert -+--\right\rangle$		*Y*_1_	*Z*_3_*Z*_4_	*Y*_1_
$\left\vert --++\right\rangle$		*X*_1_*Y*_2_	*X*_3_	*Y*_1_*Y*_5_
$\left\vert --+-\right\rangle$		*Y*_2_	*Z*_3_*Y*_4_	*Z*_1_*Y*_5_
$\left\vert ---+\right\rangle$		*Y*_1_*Z*_2_	*Y*_3_	*Z*_1_*Z*_5_
$\left\vert ----\right\rangle$		*Z*_1_*Z*_2_	*Y*_4_	*Y*_1_*Z*_5_

Corrections to the 5 data qubits to project them back into the logical codespace of the 5-qubit code depending on the ancilla outcomes and which data qubit was lost.

Note that for erasure correction, we do not use the error-correction protocol with the flag qubit since we found that it does not improve the fidelity of the error-corrected state compared to not using the flag qubit. This is due to the fact that the effectiveness of the flag qubit protocol relies on the initial 5-qubit logical state having at most a Pauli error on 1 physical qubit, while an erasure error effectively induces a correlated error, i.e., it is not merely a single-qubit Pauli error. For how the erasure errors are taken into account in the effective error rate, we refer to Supplementary Note 9.

### Error model

We model the noise in the two-qubit gates between two local spins using the two-qubit depolarizing channel

5 $${{{\Lambda }}}_{2}(\epsilon )=(1-\epsilon )\varrho +\frac{\epsilon }{15}\mathop{\sum}\limits_{P\in {\{I,X,Y,Z\}}^{\otimes 2}\setminus {I}^{\otimes 2}}P\varrho P.$$

where *ϵ* is a general error rate and *ϱ* is a two-qubit density matrix. In our model, a non-teleported two-qubit gate is followed by Λ_2_(*ϵ*_0_). Conversely, a teleported two-qubit gate, as shown in Fig. 5, involves 3 two-qubit gates that each have an error of *ϵ*_0_. Thus, we consider that an error of Λ_2_(3*ϵ*_0_) follows immediately after a teleported two-qubit gate since *ϵ*_0_ ≪ 1.

**Fig. 10:**
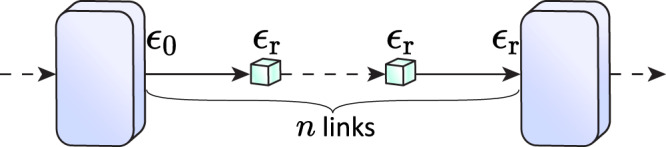
Error propagation in the network. Error propagated by the repeater operations between two consecutive TYPE II nodes with *n* (parallel) links in between. Only 1 out of 5 of the parallel links is shown for simplicity.

Another noise channel that we consider is the single-qubit depolarizing channel, which is given by

6 $${{\Lambda }}(\epsilon )=(1-\epsilon )\rho +\frac{\epsilon }{3}\mathop{\sum}\limits_{P\in \{X,Y,Z\}}P\rho P,$$

where *ρ* is a single-qubit density matrix. As previously noted in the section “Quantum-repeater protocol”, each of the 5 logical qubits at the tree-code level in the transmission is subjected to such error channel with an error rate of *ϵ*_trans_, i.e., Λ(*ϵ*_trans_). The expression for the transmission error is given by

7 $${\epsilon }_{{{{\rm{trans}}}}}=1-{(1-{\epsilon }_{{{{\rm{r}}}}})}^{n}(1-{\epsilon }_{0}).$$

where *ϵ*_r_ is the re-encoding error and *n* is the number of links between consecutive TYPE II nodes as illustrated in Fig. 10. Note that we have parameterized the number of TYPE I nodes as *m*_I_ = *m*_II_(*n* − 1). We consider the error induced by both the tree decoding and encoding step in TYPE II nodes. The tree encoding step in TYPE II nodes introduces an error of *ϵ*_0_. Conversely, the decoding step at a TYPE I and TYPE II node is similar, hence it introduces an error of *ϵ*_r_. However, instead of decoding the tree upon reception into a fresh tree, TYPE II nodes would perform a SWAP gate, which we assume has an error of *ϵ*_0_, between the decoded qubit from a tree and an auxiliary memory spin in preparation for a teleported two-qubit gate for the 5-qubit code stabilizer operations as detailed in the section “Implementation”.

Regarding the tree generation scheme from ref. ^17^ which we consider in our work, we note that, in principle, it could lead to correlated errors in the tree-cluster states since it involves repeated photon emission from the same emitter with an error rate of *ϵ*_0_. This error on the emitter thus might propagate to more than a single photon in the tree. It is, however, outside the scope of this work to consider the effect of such correlated errors. We note that they could potentially be kept small by, e.g., intermediate error detection steps in the generation procedure^32^. We assume that we are able to suppress the correlated errors, and then we can say that each photon in our trees is merely subjected to Λ(*ϵ*_0_). From our numerical simulation of the tree re-encoding procedure, we find that our model results in a re-encoding error rate of *ϵ*_r_ ≈ 3*ϵ*_0_ at each TYPE I node for the tree sizes considered in this work (see Supplementary Note 10). This reflects the fact that despite the tree-clusters consisting of hundreds of photons each subjected to Λ(*ϵ*_0_), the dominant error comes from errors on the first-level photon and spin-qubit involved in the re-encoding step. Errors on the remaining photons, which are measured out, are efficiently suppressed by employing a majority-voting correction strategy.

### Repeater performance

The key performance metrics of the repeater network that we consider are the end-to-end transmission probability of the message qubit and the quality of the transmitted qubit. To characterize the end-to-end transmission probability of the message qubit, we start by considering the transmission probability, *η*_e_, of a single tree-cluster state with branching vector **t** = [*b*_0_, *b*_1_, …, *b*_*d*_] between consecutive repeater nodes. From ref. ^35^, we have that

8 $${\eta }_{{{{\rm{e}}}}}=[{(1-\mu +\mu {R}_{1})}^{{b}_{0}}-{(\mu {R}_{1})}^{{b}_{0}}]{(1-\mu +\mu {R}_{2})}^{{b}_{1}},$$

where ${R}_{k}=1-{[1-(1-\mu ){(1-\mu +\mu {R}_{k+2})}^{{b}_{k+1}}]}^{{b}_{k}}$ with *R*_*d*+1_ = 0, *b*_*d*+1_ = 0, and *μ* = 1 − *ηη*_d_. Here, $\eta =\exp (-{L}_{0}/{L}_{{{{\rm{att}}}}})$ is the transmission probability of a single photon between repeater nodes with *L*_0_ being the inter-node distance and *L*_att_ being the attenuation length of the optical fiber. Furthermore, *η*_d_ is the combined efficiency of in/out-coupling of the photon, frequency conversion, and photon detection. We assume that efficient frequency conversion to the telecom band is possible such that *L*_att_ = 20 km.

Without erasure-error correction on the 5-qubit code level, the end-to-end transmission probability of the message qubit would be given by ${\eta }_{{{{\rm{e}}}}}^{5{m}_{{{{\rm{tot}}}}}}$ where *m*_tot_ = *m*_I_ + *m*_II_ is the total number of nodes in the repeater excluding the start node with *m*_I_ (*m*_II_) being the number of TYPE I (TYPE II) nodes in the network. Note that we exclude the start node but include the end node when counting *m*_II_. Nonetheless, it is advantageous to leverage the outer 5-qubit code to also correct for erasure errors. Thus, we need to consider how this affects the end-to-end transmission probability of the message qubit.

We choose to treat cases with more than one erasure error, i.e., more than one failed tree encoding, at the same TYPE II node as a failed transmission for simplicity as mentioned in the section “Erasure-error correction”. The total transmission probability in this case can then be expressed as

9 $$\mathop{\sum }\limits_{i=0}^{{m}_{{{{\rm{II}}}}}}\left(\begin{array}{c}{m}_{{{{\rm{II}}}}}\\ i\end{array}\right){p}_{{{{\rm{trans}}}}}({m}_{{{{\rm{II}}}}},i),$$

with

10 $${p}_{{{{\rm{trans}}}}}({m}_{{{{\rm{II}}}}},i)={[{\eta }_{{{{\rm{e}}}}}^{5n}]}^{{m}_{{{{\rm{II}}}}}-i}{[5{\eta }_{{{{\rm{e}}}}}^{4n}(1-{\eta }_{{{{\rm{e}}}}}^{n})]}^{i},\quad {m}_{{{{\rm{II}}}}}\ge i,$$

being the probability of successful transmission with 1-erasure errors in *i* distinct TYPE II nodes. Besides the transmission probability, we also need to assess the quality of the transmitted qubits. To this end, we consider a scenario where the transmitted qubits are used to distill a secret key. In particular, we assume that the six-state protocol^49^ is employed, which allows us to quantify the quality of the transmitted qubits using its asymptotic secret key fraction. This is given by^52^

11 $${f}_{{m}_{{{{\rm{II}}}}},i}=\max \left((1-Q)\left[1-h\left(\frac{1-3Q/2}{1-Q}\right)\right]-h(Q),0\right),$$

where $h(x)=-x{\log }_{2}x-(1-x){\log }_{2}(1-x)$ is the binary entropy and

12 $$Q=2{\epsilon }_{{{{\rm{eff}}}}}({m}_{{{{\rm{II}}}}},i)/3,$$

denotes the QBER (QuBit Error Rate) of the fully decoded qubit at the end node. The quantity *ϵ*_eff_(*m*_II_, *i*) is the effective error rate of the received qubit at the end node given there were *i* erasure-error occurrences and *m*_II_ TYPE II nodes.

With our error model, we determine *ϵ*_eff_(*m*_II_, *i*) from numerical simulations of the 5-qubit code throughout the repeater network. The details of these simulations can be found in Supplementary Note 7 where we also provide a semi-analytical approximation to *ϵ*_eff_(*m*_II_, *i*), which matches the numerical results to great precision for the relevant range of effective error rates, i.e., error rate that is less than the threshold QBER of the associated QKD protocol.

### Numerical minimization

We minimize the cost function *C* in eq. (4) with respect to *L*_0_, *m*_II_, and **t** with the following constraints: minimum inter-repeater distance *L*_0_ ≥ 1 km, the maximum number of TYPE II nodes never exceeds half of the total number of nodes, i.e., *m*_II_ ≤ ⌊*m*_tot_/2⌋, the maximum photon number ${N}_{\max }=300$ with the total photon number $N=\mathop{\sum }\nolimits_{i = 0}^{d}\mathop{\prod }\nolimits_{j = 0}^{i}{b}_{j}$ and a fixed depth of the tree *d* = 2. Then, the minimization of *C* can be written as

13 $$\begin{array}{l}{C}_{\min }\,=\,\mathop{\min }\limits_{{L}_{0},{m}_{{{{\rm{II}}}}},{{{\bf{t}}}}}C,\,{{\mbox{subject to}}}\,:\\\qquad\qquad {L}_{0}\ge 1\,{{{\rm{km}}}},\\\qquad\qquad 1\le {m}_{{{{\rm{II}}}}}\le \lfloor {m}_{{{{\rm{tot}}}}}/2\rfloor ,\\\qquad\qquad {{{\bf{t}}}}\in \{[{b}_{0},{b}_{1},{b}_{2}]\,| \,\forall j,{b}_{j}\in {{\mathbb{Z}}}_{\ > \ 0}\,\,{{\mbox{and}}}\,\,N\le {N}_{\max }\},\end{array}$$

where ${{\mathbb{Z}}}_{\ > \ 0}$ is the set of all positive integers. We chose the quantity ⌊*m*_tot_/2⌋ as the maximum number of TYPE II nodes in our optimization since the approximations that make eq. (1) accurate starts to break down beyond this value. This is because we can no longer properly place the TYPE II nodes such that they are evenly spaced beyond this value (see Supplementary Note 8). The rest of the parameters are fixed and their values are shown in Table 1. To ensure that the true optimum does not lie in a regime where only TYPE II nodes are permitted in the network, we independently optimized such a configuration with respect to the cost function and found that both the resulting secret key rate and cost are significantly worse than for the hybrid configuration (see Supplementary Note 6).

### Supplementary information

Supplementary Information: Resource-efficient fault-tolerant one-way quantum repeater with code concatenation

## Data Availability

The generated data in this study can be found at 10.4121/b9c7327e-97b2-4ea2-9b74-18c51f265027.v1.

## Code availability

The code used for obtaining the presented numerical results is available at https://github.com/bernwo/code-concatenated-quantum-repeater.
