# Supplementary material for: Resource-efficient fault-tolerant one-way quantum repeater with code concatenation
Source: npj Quantum Inf. 2023 Dec 12;9(1):123. doi: 10.1038/s41534-023-00792-8 (PMC11041798; doi:10.1038/s41534-023-00792-8)
Supplement: Supplementary file 1 — Supplementary Information: Resource-efficient fault-tolerant one-way quantum repeater with code concatenation [file 41534_2023_792_MOESM1_ESM.pdf]

Supplementary Information: Resource efficient fault-tolerant one-way quantum repeater with code concatenation

SUPPLEMENTARY NOTE 1. ERROR/ERASURE CORRECTION PROTOCOL IN A TYPE II NODE

As explained in the main text, a message qubit in some arbitrary state is first encoded into a logical qubit on the 5-qubit code level, which comprises 5 physical spin qubits. Each of those 5 physical spin qubits is then encoded onto a tree cluster state which are sent along the repeater network in parallel across 5 sets of optical fibers. The repeater network consists of both TYPE I and TYPE II nodes. Once the 5 sets of trees arrive at a TYPE II node, it performs quantum error correction at the 5-qubit code level in a fault-tolerant manner via flag qubits. However, if a tree happens to be completely unrecoverable, i.e., more than 50% of the photons in the tree were lost in transmission, then quantum erasure correction, which does not utilize the flag qubits, is performed instead. The protocol is shown as a flowchart in Supplementary Figure 1 below.

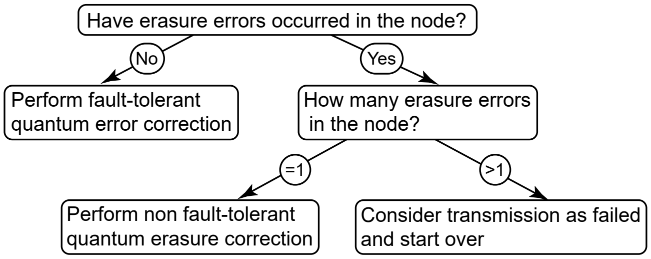

Supplementary Figure 1. The control flow diagram of how a TYPE II node decides whether to perform quantum error correction or quantum erasure correction.

As shown in Supplementary Figure 1, the TYPE II node performs the non fault-tolerant quantum erasure correction when a 1-erasure error is heralded. This happens with probability $5\eta_e^{4n}(1-\eta_e^n)$, where η_e is the transmission probability of a tree from one node to the next and $n-1$ is the number of TYPE I nodes between consecutive TYPE II nodes as explained in the main text. However, for k-erasure errors with $k > 1$, we abort and restart the transmission of the message qubit. This happens with probability $1 - \eta_e^{5n} - 5\eta_e^{4n}(1 - \eta_e^n)$. In theory, a TYPE II node operating under the 5-qubit code is also capable of correcting for 2-erasure errors as explained in the main text. We did not consider this in our secret key rate analysis because the probability of two trees being unrecoverable at the same TYPE II node is negligible compared to the probability for a 1-erasure error. To show this, we first note that for the data points where the secret key rate of the hybrid repeater scheme is higher than that of the homogeneous repeater

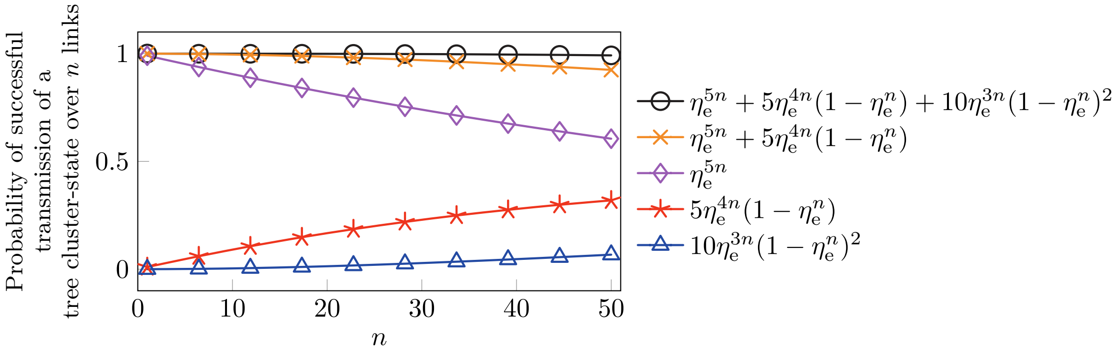

Supplementary Figure 2. The probabilities of successful transmission of a tree cluster-state over n links as a function of n. The transmission probability of a tree cluster-state over 1 link is given by η_e (see main text). The circle symbol denotes the total successful transmission probability assuming that both the 1- and 2-erasure errors are being corrected. The cross symbol denotes the total successful transmission probability assuming that only the 1-erasure errors are being corrected. The contributions to the total probability are shown by the diamond, star, and triangle symbols which denote the probability of no trees being lost, one tree being lost, and two trees being lost, respectively. We fixed the efficiency to $\eta_e = 0.998$, which is the typical value found in our numerical optimization.

scheme (see main text) and $\epsilon_{\rm r} \in \{0.3‰, 0.5‰, 0.1\%, 0.2\%\}$, the typical number of links is found to be in the range of $1 \lesssim n \lesssim 50$. Plotting the individual contributing terms to the total successful transmission probability for this range in Supplementary Figure 2 reveals that the contributing term from the 2-erasure error correction is indeed negligible. Note that this term is also negligible for when $\epsilon_{\rm r} = 0.1‰$ where the corresponding number of links found is $50 \lesssim n \lesssim 150$ because the $\eta_{\rm e}$ in this regime is much closer to unity compared to other higher re-encoding error probabilities. By omitting the contribution from correcting 2-erasure errors, we are slightly underestimating our secret key rate.

SUPPLEMENTARY NOTE 2. FAULT-TOLERANT ERROR CORRECTION PROTOCOL

Here, we explain the details of the fault-tolerant quantum error correction protocol from Ref. [1] as noted in both the main text and Supplementary Figure 1. The quantum circuit corresponding to the fault-tolerant 5-qubit code error correction is shown in Supplementary Figure 3a along with the sub-circuits labelled according to their respective stabilizers, which as a reminder are $X_1Z_2Z_3X_4$, $X_2Z_3Z_4X_5$, $X_1X_3Z_4Z_5$, and $Z_1X_2X_4Z_5$. The protocol for the fault-tolerant 5-qubit code error correction is shown explicitly in definition 1. This protocol enables us to suppress weight $w = 2$ errors which otherwise would have caused undetectable logical errors. The protocol involves exiting from the fault-tolerant circuit under certain conditions, and changing into the non fault-tolerant circuit, i.e., the 5-qubit code circuit without the flag qubit, to extract the syndrome from the ancilla qubits. To differentiate the syndrome extracted from the fault-tolerant circuit from the non fault-tolerant circuit, we label the syndrome extracted from the non fault-tolerant circuit with the "$\prime$" sign, i.e., A'_k, which represents the value of the $k^{\rm th}$ measured ancilla qubit.

> **Definition 1.** The protocol for the fault-tolerant 5-qubit code is as follows:
> Begin with the fault-tolerant circuit in Supplementary Figure 3 and proceed to performing the sub-circuits in order. Depending on the measurement outcome at each sub-circuit denoted by g_k for $k \in \{1,2,3,4\}$, one would need to make the following decisions:
>
> 1. If $F_k = A_k = +1$, i.e., flag and ancilla are not triggered, then continue using the fault-tolerant circuit and extract A_{k+1} and A_{k+1}. If all four flag qubits and four ancilla qubits were not triggered, we are finished with no corrections needed.
> 2. If $F_k = +1$ and $A_k = -1$, i.e., flag is not triggered while ancilla is triggered, then switch to the non fault-tolerant circuit and measure A'_1, A'_2, A'_3, A'_4. Finish by applying the weight $w \leq 1$ corrections shown in Supplementary Figure 3b.
> 3. If $F_k = -1$ and $A_k = \pm 1$, i.e., flag is triggered regardless of syndrome outcome, then switch to the non fault-tolerant circuit and measure A'_1, A'_2, A'_3, A'_4. Finish by applying the weight $w \leq 2$ corrections shown in Supplementary Figure 3c.

(a)

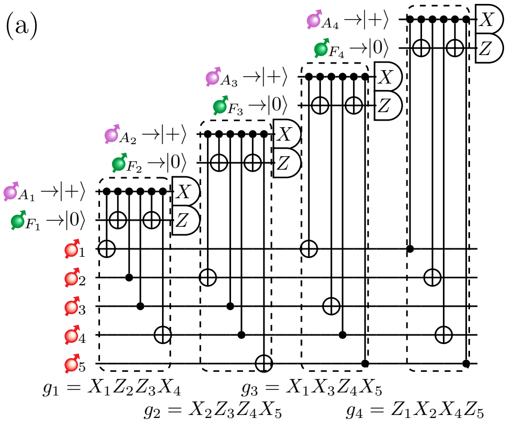

(b)

A'_1	A'_2	A'_3	A'_4	Correction
+1	+1	+1	+1	I
+1	+1	+1	−1	X_1
+1	+1	−1	+1	Z_3
+1	+1	−1	−1	X_5
+1	−1	+1	+1	Z_5
+1	−1	+1	−1	Z_2
+1	−1	−1	+1	X_4
+1	−1	−1	−1	Y_5
−1	+1	+1	+1	X_2
−1	+1	+1	−1	Z_4
−1	+1	−1	+1	Z_1
−1	+1	−1	−1	Y_1
−1	−1	+1	+1	X_3
−1	−1	+1	−1	Y_2
−1	−1	−1	+1	Y_3
−1	−1	−1	−1	Y_4

(c)

				Correction				
A'_1	A'_2	A'_3	A'_4	Triggered:	F_1	F_2	F_3	F_4
+1	+1	+1	+1		I	I	I	I
+1	+1	+1	−1		X_1	X_1	X_1	X_1
+1	+1	−1	+1		Z_3	Z_3	X_4Z_5	X_4Z_5
+1	+1	−1	−1		X_5	X_5	X_1Z_3	X_5
+1	−1	+1	+1		Z_3X_4	X_2X_3	Z_5	Z_5
+1	−1	+1	−1		Z_2	X_4X_5	Z_2	Z_2
+1	−1	−1	+1		X_4	X_1Y_5	X_4	X_4
+1	−1	−1	−1		Y_5	Y_5	Y_5	Z_1Y_2
−1	+1	+1	+1		Y_3X_4	X_2	X_2	X_2
−1	+1	+1	−1		X_1X_2	Z_4	Z_4	Z_4
−1	+1	−1	+1		X_3X_4	Z_4X_5	Z_1	Z_1
−1	+1	−1	−1		Y_1	Y_1	Y_4Z_5	Y_4Z_5
−1	−1	+1	+1		X_1Y_2	Y_4X_5	X_3	X_3
−1	−1	+1	−1		Y_2	Y_2	Z_4Z_5	Z_4Z_5
−1	−1	−1	+1		Y_3	Y_3	Y_3	Y_3
−1	−1	−1	−1		Y_4	Y_4	X_1Y_3	Z_1Z_2

Supplementary Figure 3. (a) The fault-tolerant 5-qubit code quantum circuit with flag qubit. The same ancilla (flag) spin is re-initialized in state $|+\rangle$ ($|0\rangle$) before being sequentially measured at each stabilizer g_k for $k \in \{1,2,3,4\}$. Sections of the circuit, i.e., sub-circuits, are boxed and labelled according to their respective stabilizers g_k acting on the data qubits j with $j \in \{1,2,3,4,5\}$. (b) Correction look-up table of weight $w \leq 1$ and (c) table of weight $w \leq 2$.

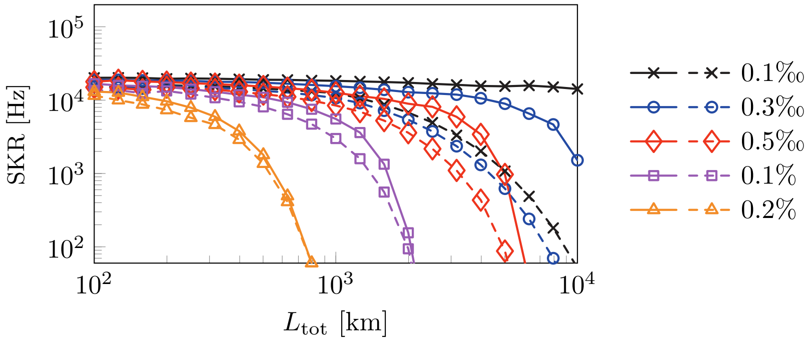

Supplementary Figure 4. The secret key rate SKR of the hybrid repeater network for different values of re-encoding error rates ϵ_{r} optimized with respect to the cost function shown in the main text. The solid and dashed lines represent the SKR values when the 5-qubit code is and is not leveraged for erasure error correction, respectively.

SUPPLEMENTARY NOTE 3. EFFECT OF ERASURE CORRECTION ON THE 5-QUBIT CODE LEVEL

In this section, we show that by leveraging the 5-qubit code to additionally correct for erasure errors, we obtain a substantial increase in the resulting optimized secret key rate. This increase is shown in Supplementary Figure 4. We can also see that the higher re-encoding errors limit the potential for improved secret key rates. This is expected because for a 1-erasure error correction to succeed, it is imperative that the rest of the qubits are error free. Once the error rate is too high, we lose the advantage of 1-erasure error correction.

SUPPLEMENTARY NOTE 4. PROCESSING TIME IN A TYPE II NODE

To quantify the secret key rate of the network, it is important to consider the processing time per repeater node. Since a TYPE II node would always take longer than a TYPE I node for processing and become the processing bottleneck, we analyze only the processing time of a TYPE II node. Let p_j represent the probability that either the ancilla or the flag qubit is detected as triggered in the readout of the jth sub-circuit in Supplementary Figure 3a, and p_5 represents the probability that no ancilla and flag qubits were triggered in the entirety of the fault-tolerant circuit. Note that $\sum_{j=1}^{5} p_j = 1$ must be true. Then, the act of performing fault-tolerant quantum error correction takes a time

$$T_{\text{f}} = p_5 \sum_{i=1}^{4} \tau_{\text{f}_i} + \sum_{k=1}^{4} p_k \Big(\tau_{\text{nf}} + \sum_{l=1}^{k} \tau_{\text{f}_l} \Big), \tag{1}$$

where $\tau_{\text{nf}} = 3\tau_{\text{ss}} + 13\tau_{\text{tele}} + 4\tau_{\text{meas}}$ is the time required to perform the non fault-tolerant circuit while τ_{f_k} is the time needed to perform the sub-circuits shown in Supplementary Figure 3a, and they are given by

$$\begin{aligned} \tau_{\text{f}_1} &= 3\tau_{\text{ss}} + 3\tau_{\text{tele}} + \tau_{\text{meas}}, \qquad & \tau_{\text{f}_2} &= 2\tau_{\text{ss}} + 4\tau_{\text{tele}} + \tau_{\text{meas}}, \\ \tau_{\text{f}_3} &= 3\tau_{\text{ss}} + 3\tau_{\text{tele}} + \tau_{\text{meas}}, \qquad & \tau_{\text{f}_4} &= 3\tau_{\text{ss}} + 3\tau_{\text{tele}} + \tau_{\text{meas}}. \end{aligned} \tag{2}$$

It follows then that the total time to perform all subcircuits is $\sum_{l=1}^{4} \tau_{\text{f}_l} = 11\tau_{\text{ss}} + 13\tau_{\text{tele}} + 4\tau_{\text{meas}}$. Note that the maximum of T_{f} occurs when $p_4 = 1$. This corresponds to the situation in which no subcircuits were skipped in Supplementary Figure 3a. If any TYPE II node takes this long for processing, then the rest of the nodes in the network need to wait for just as long, thus it becomes the bottleneck of the network processing time. It is this time that we consider in the total processing time of repeater nodes in the network together with the tree generation time τ_{tree}, which is explained in the main text. The total processing time per node is thus given by

$$\begin{aligned} \tau_{\text{tot}} &= \tau_{\text{tree}} + \tau_{\text{nf}} + \sum_{l=1}^{4} \tau_{\text{f}_l}, \\ &= \tau_{\text{tree}} + 14\tau_{\text{ss}} + 26\tau_{\text{tele}} + 8\tau_{\text{meas}}. \end{aligned} \tag{3}$$

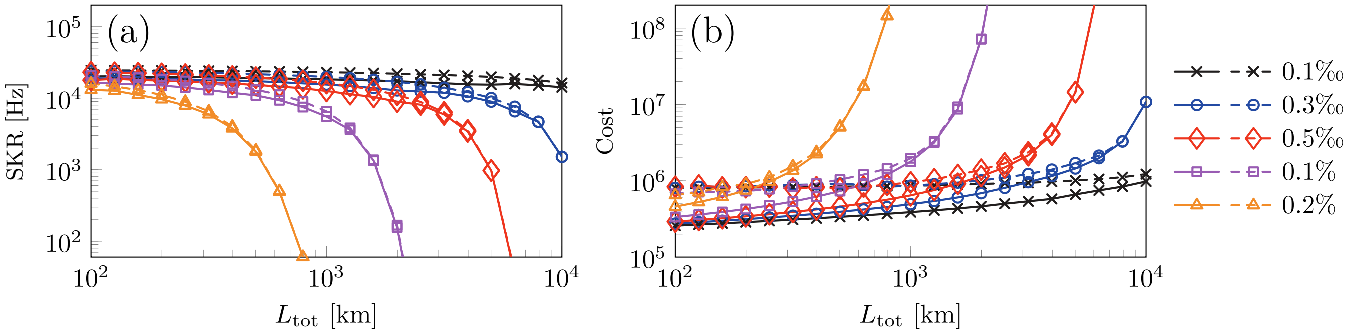

Supplementary Figure 5. (a) The secret key rate SKR of the hybrid repeater network with $\kappa = 1$ for the configurations in which the secret key rate is optimized with respect to the cost function (solid lines) and purely maximized (dashed lines). (b) The corresponding cost.

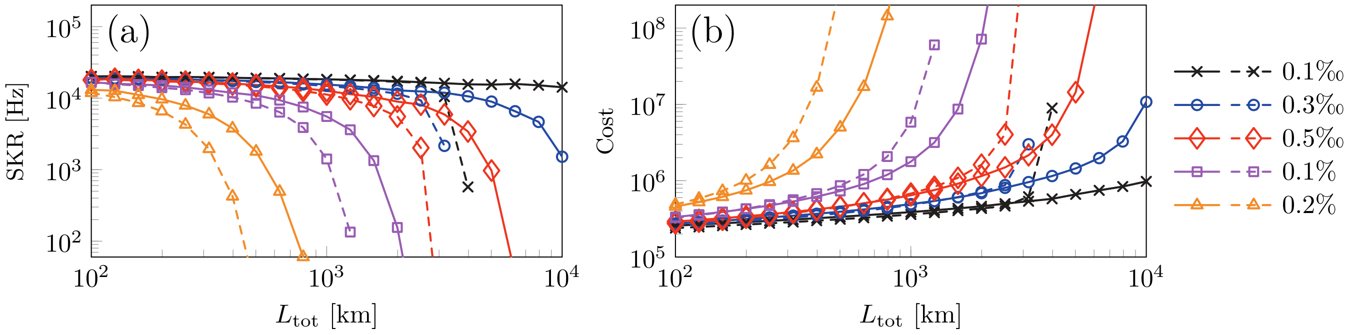

Supplementary Figure 6. (a) The secret key rate SKR of the hybrid repeater network with $\kappa = 1$ for the configurations in which both types of nodes are allowed (solid lines) and only TYPE II nodes are allowed (dashed lines). (b) The corresponding cost.

SUPPLEMENTARY NOTE 5. MAXIMIZED SECRET KEY RATE

In this section, we discuss the difference of the secret key rate and cost between maximizing for the secret key rate (dashed lines) and optimizing the network configuration with respect to the cost function (solid lines) shown in Supplementary Figure 5. In particular, we showed that in Supplementary Figure 5a that the discrepancy in the secret key rate is small between the two configurations, yet the cost is significantly higher for the configuration in which the secret key rate is maximized in Supplementary Figure 5b, especially at shorter distances. This suggests that by merely optimizing the network with respect to the cost function, we can achieve near-optimum secret key rates at the benefit of significantly reduced resource requirement. The secret key rate and cost converges however due to the increasing total distance, which limits the number of loss errors which can be corrected in the network.

SUPPLEMENTARY NOTE 6. HAVING ONLY TYPE II NODES IN THE REPEATER NETWORK

In this section, we show via Supplementary Figure 6 that having only TYPE II nodes in the network is not an optimal approach, despite optimizing for this configuration independently with respect to the cost function noted in the main text. Specifically, the resulting secret key rate in Supplementary Figure 6a is performing worse if we permit only TYPE II nodes in the network. The performance discrepancy is especially apparently at larger total distances, where loss errors are likely. This inadvertently causes the cost in Supplementary Figure 6b to be higher. This result implies that a code concatenated network in which only TYPE II nodes is a configuration that is far from being ideal, especially at larger total distances.

SUPPLEMENTARY NOTE 7. APPROXIMATING FIDELITY VIA RECURSION

To calculate the resulting fidelity of our message qubit at the end node exactly, one can start with a density matrix of the error-free 5-qubit code logical state, followed by the application of the stabilizer operations for each TYPE II node in the network, effectively evolving the density matrix analytically. As noted in fig. 8 in the main text, however, there can be up to hundreds of TYPE II nodes in the network, which makes the calculation a challenging task computationally. In this section, we introduce a method to approximate this fidelity up to great precision by exploiting the symmetry of the network.

Let ρ_0 be the 5-qubit density matrix that describes the 5-qubit code logical state, free from any errors, and $\rho_\perp$ is a matrix, which attempts to describe the state that is orthogonal to the logical state. The qubits in the network are subjected to errors in transmission, which is why a TYPE II node performs (fault-tolerant) quantum error correction upon the reception of an incoming tree (as discussed in Supplementary Note 1). The effect of the errors introduced in the transmission and the quantum error correction performed by a TYPE II node on the message qubit can be modelled as a quantum channel acting on the density matrix of the perfect logical state

$$\mathcal{C}(\rho_0) = \alpha_1\rho_0 + \beta_1\rho_\perp, \tag{4}$$

where α_1 is the fidelity, $\beta_1 = 1 - \alpha_1$, and the matrix $\rho_\perp$ is modelled as $\rho_\perp = (\mathcal{C}(\rho_0) - \alpha_1\rho_0)/\beta_1$. As explained in the main text, we consider the nodes to be equidistant from each other regardless of their type. This means that we can apply the quantum channel in eq. (4) repeatedly, which reflects the fact that there are multiple TYPE II nodes in the network with the same number of TYPE I nodes between them. We model also the application of the quantum channel on the orthogonal state as

$$\mathcal{C}(\rho_\perp) = \alpha'\rho_0 + \beta'\rho'_\perp. \tag{5}$$

By using eqs. (4) and (5), we can obtain a *recurrence relation* below. As an approximation used in obtaining the recurrence relation, we assume that the correction on $\rho_\perp$ is highly efficient, i.e., $\alpha' \approx 1$, and therefore we only keep terms that lead to non-zero fidelity while we discard terms with β'. Applying these rules to all stages of the protocol leads to

$$\begin{aligned}
\rho_1 &= \mathcal{C}(\rho_0) = \alpha_1\rho_0 + \beta_1\rho_\perp, \\
\rho_2 &= \mathcal{C}(\rho_1) = \alpha_1\mathcal{C}(\rho_0) + \beta_1\mathcal{C}(\rho_\perp) = \alpha_2\rho_0 + \beta_2\rho_\perp, \\
&\vdots \\
\rho_{m_{\mathrm{II}}} &= \mathcal{C}(\rho_{m_{\mathrm{II}}-1}) = \alpha_{m_{\mathrm{II}}}\rho_0 + \beta_{m_{\mathrm{II}}}\rho_\perp,
\end{aligned} \tag{6}$$

where $\alpha_{m_{\mathrm{II}}}$ and $\beta_{m_{\mathrm{II}}}$ are given by

$$\alpha_{m_{\mathrm{II}}} = \alpha_1\alpha_{m_{\mathrm{II}}-1} + \beta_{m_{\mathrm{II}}-1}\alpha', \quad \beta_{m_{\mathrm{II}}} = \beta_1\alpha_{m_{\mathrm{II}}-1}. \tag{7}$$

From here, we obtain

$$\alpha_{m_{\mathrm{II}}} = \alpha_{m_{\mathrm{II}}-1}\alpha_1 + \alpha_{m_{\mathrm{II}}-2}(1-\alpha_1)\alpha', \text{ with } \alpha_i = \begin{cases} \alpha_1, & \text{if } i = 1 \\ 1, & \text{if } i = 0 \\ 0, & \text{if } i < 0 \end{cases}. \tag{8}$$

Finally, solving the recurrence relation in eq. (8) yields

$$\boxed{\mathcal{F}_{m_{\mathrm{II}},n} \equiv \alpha_{m_{\mathrm{II}}} \equiv \frac{(\alpha_1+\zeta)^{m_{\mathrm{II}}+1} - (\alpha_1-\zeta)^{m_{\mathrm{II}}+1}}{2^{m_{\mathrm{II}}+1}\zeta}, \quad m_{\mathrm{II}} \geq 1,} \tag{9}$$

where $\zeta = \sqrt{\alpha_1^2 + 4(1-\alpha_1)\alpha'}$ and $\mathcal{F}_{m_{\mathrm{II}},n}$ is the approximated fidelity for m_{II} TYPE II nodes and $n-1$ TYPE I nodes between consecutive TYPE II nodes. Note that the quantum channel $\mathcal{C}$ depends on n and thus α_1. Hence, we label the approximated fidelity with the subscript n in eq. (9). The term α' can be expressed in terms of α_1 and α_2 by noting the expressions ρ_1 and ρ_2 in eq. (6):

$$\mathcal{C}(\rho_\perp) = \frac{\rho_2 - \alpha_1\rho_1}{\beta_1} = \frac{\alpha_2 + \alpha_1^2}{\beta_1}\rho_0 + \cdots. \tag{10}$$

By comparing eqs. (5) and (10), we find that $\alpha' = (\alpha_2 + \alpha_1^2)/\beta_1$, and therefore we can express ζ in eq. (9) as $\zeta = \sqrt{4\alpha_2 - 3\alpha_1^2}$. We now have an efficient manner to obtain an approximated fidelity for $m_{\mathrm{II}} > 2$ by computing α_1 and α_2. Note that $\mathcal{F}_{m_{\mathrm{II}},n}$ as shown in eq. (9) is exact for $m_{\mathrm{II}} = 1$ and $m_{\mathrm{II}} = 2$. Then, we can define the effective error as

$$\epsilon_{\mathrm{eff}} = 1 - \mathcal{F}_{m_{\mathrm{II}},n}. \tag{11}$$

Note that if we set $\alpha' = 0$, we effectively arrive at the naïve/pessimistic approximation, where the effective error simply becomes $1 - \alpha_1^{m_{\mathrm{II}}}$ shown in Supplementary Figure 7. In Supplementary Figure 7, we show that the approximation of using the recurrence relation (orange dashed line) yields remarkably accurate results compared to the exact effective error rate (solid black line) in the regime of interest, i.e., $\epsilon_{\mathrm{eff}} \lesssim 12.61\%$ because 12.61% is approximately the threshold QBER for generating secret keys in the six-state quantum key distribution protocol [2], whereas the naïve approximation (red dotted line) yields effective error rates about an order of magnitude higher than the exact result.

If erasure errors are also present, it is no longer feasible to only use the effective error in eq. (11) because we the channels are no longer the same throughout the network. However, we can simply modify the expression to include correcting for i 1-erasure errors in the network

$$\epsilon_{\mathrm{eff}}(m_{\mathrm{II}}, i) = 1 - (\mathcal{F}_{m_{\mathrm{II}}-i,n})(1 - \epsilon_{\mathrm{loss},n})^i. \tag{12}$$

where $\epsilon_{\mathrm{loss},n}$ is defined as one minus the fidelity of the logical qubit at the 5-qubit code level at a TYPE II node after a 1-erasure correction with the condition that the logical qubit was error-free in the previous TYPE II node with n links between them. Note that it is implied in eq. (12) that our message qubit cannot be recovered after 1 failed attempt at erasure correction at the 5-qubit code level. This is a reasonable assumption since erasure correction is vulnerable to additional errors on the intact-qubits.

SUPPLEMENTARY NOTE 8. UNEVEN DISTANCE BETWEEN TYPE II NODES

It is not always possible to place TYPE II nodes in the network such that they are evenly spaced between each other for a given total distance L_{tot}. Here, we explain the specific strategy we employed in the main text for placing the repeater nodes as evenly as possible.

For some given total distance L_{tot} we can calculate the total number of nodes in the network (excluding the start node) as

$$m_{\mathrm{tot}} = \frac{L_{\mathrm{tot}}}{L_0}, \tag{13}$$

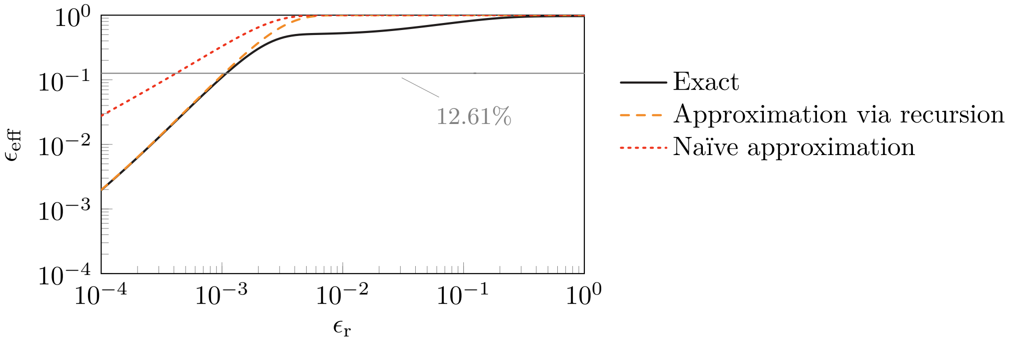

Supplementary Figure 7. The effective error rate ϵ_{eff} as a function of the re-encoding error ϵ_{r}. The effective error rate was calculated exactly for $n = 8$, $m_{\mathrm{II}} = 125$, assuming no erasure errors. The effective error rates are approximated using both the recursion method and naïve method.

where L_0 is the distance between consecutive nodes. Note that for convenience, we choose the values of L_0 such that $m_{\mathrm{tot}} \in \mathbb{Z}_{>0}$. Given no erasure errors in the network, the effective error rate of the message qubit received at the end node is

$$\epsilon_{\mathrm{eff}}(m_{\mathrm{II}}, 0) = 1 - (\mathcal{F}_{m_{\mathrm{II}}-1,n'})(\mathcal{F}_{1,n''}), \tag{14}$$

where $n' = \lfloor m_{\mathrm{tot}}/m_{\mathrm{II}} \rfloor$ and $n'' = m_{\mathrm{tot}} - n'(m_{\mathrm{II}} - 1)$ are the number of links between consecutive TYPE II nodes as shown in Supplementary Figure 8. Note that for $m_{\mathrm{II}} > \lfloor m_{\mathrm{tot}}/2 \rfloor$, the repeater nodes can no longer be placed reasonably evenly, thus we constrained our numerical minimization of the cost function to $m_{\mathrm{II}} \leq \lfloor m_{\mathrm{tot}}/2 \rfloor$ as noted in Methods section the main text. This is extended for non-zero erasure errors in Supplementary Note 9.

SUPPLEMENTARY NOTE 9. ACCOUNTING FOR ERASURE ERRORS IN THE NETWORK

Here, we modify eq. (14) to account for erasure error correction:

$$\epsilon_{\mathrm{eff}}(m_{\mathrm{II}}, i) \approx \begin{cases} 1 - (1 - \epsilon_{\mathrm{loss},n})^i (\mathcal{F}_{m_{\mathrm{II}}-i, m_{\mathrm{tot}}/m_{\mathrm{II}}}) & \text{, if } (m_{\mathrm{tot}} \bmod m_{\mathrm{II}} = 0) \\ 1 - (1 - \epsilon_{\mathrm{loss},n'})^i (\mathcal{F}_{m_{\mathrm{II}}-1-i,n'})(\mathcal{F}_{1,n''}) & \text{, if } (m_{\mathrm{tot}} \bmod m_{\mathrm{II}} > 0) \wedge (m_{\mathrm{II}} - 1 \geq i) \\ 1 - (1 - \epsilon_{\mathrm{loss},n'})^{i-1} (1 - \epsilon_{\mathrm{loss},n''}) & \text{, if } (m_{\mathrm{tot}} \bmod m_{\mathrm{II}} > 0) \wedge (m_{\mathrm{II}} = i) \end{cases}, \tag{15}$$

This is the expression which is used in our numerical optimization of the secret key rate.

SUPPLEMENTARY NOTE 10. RE-ENCODING ERROR SIMULATION

In this section we determine the tree-level re-encoding error probability ϵ_{r} and find that it relates to the single-qubit error probability ϵ_0 according to

$$\epsilon_{\mathrm{r}} \approx 3\epsilon_0. \tag{16}$$

First we introduce two different (yet equivalent) definitions of the single-qubit depolarizing channel

$$\mathcal{D}_p(\rho) = p\rho + \frac{1-p}{2} I = \mathcal{D}'_\epsilon(\rho) = (1-\epsilon)\rho + \frac{\epsilon}{3}(X\rho X + Y\rho Y + Z\rho Z), \tag{17}$$

where X, Y and Z are the Pauli operators, I is the identity operator and

$$\epsilon = \frac{3}{4}(1-p). \tag{18}$$

Here, we include both definitions because the first simplifies our treatment here while the second is the one used in the main text. We model the re-encoding procedure as follows.

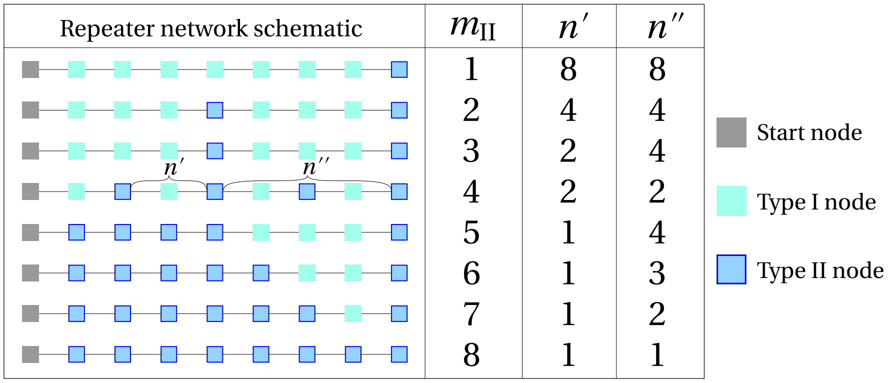

Supplementary Figure 8. Repeater network layout in the presence of TYPE I and TYPE II nodes for varying number of TYPE II nodes m_{II} and fixed number of total nodes $m_{\mathrm{tot}} = 8$. Not all values of m_{II} can divide m_{tot}, hence there will be sections in which the number of links between consecutive TYPE II nodes are different, i.e., $n' = \lfloor m_{\mathrm{tot}}/m_{\mathrm{II}} \rfloor$ versus $n'' = m_{\mathrm{tot}} - n'(m_{\mathrm{II}} - 1)$.

1. The initial state ρ_i is held by a memory qubit as result of heralded storage of the tree-encoded qubit transmitted from the previous node to the current node. A second memory qubit is the root of a fresh tree state (that is otherwise photonic) that is transmitted to the next node.

2. The two memory qubits each undergo single-qubit depolarizing channel $\mathcal{D}_{p_0}$.

3. A Bell-state measurement is performed on the two memory qubits to teleport the state ρ_i into the fresh tree-level logical qubit. Single-qubit Paulis are applied to individual photons to realize the logical Pauli operation required by the teleportation protocol as specified by the outcome of the Bell-state measurement.

4. Each of the photonic qubits is subject to both a single-qubit depolarizing channel $\mathcal{D}_{p_0}$ and a pure loss channel with loss probability $1-\eta$.

5. Heralded storage is attempted to put the transmitted tree-level logical qubit into a memory qubit again. Conditioned on success, the state held by the memory qubit is denoted ρ_f.

Here, p_0 is defined in terms of the single-qubit error probability according to $\epsilon_0 = \frac{3}{4}(1-p_0)$. We will now set out to determine ρ_f as a function of ρ_i, ϵ_0 and η.

As each of the Bell states has the property $(I \otimes P)\left|\phi\right\rangle = \pm(P \otimes I)\left|\phi\right\rangle$ for all Pauli operators P, we can "move" the depolarizing noise occurring at the second memory qubit through the measurement operators of the Bell-state measurement to the first memory qubit. Then, Step 3 is just the perfect teleportation of the state

$$\mathcal{D}_{p_0} \circ \mathcal{D}_{p_0}(\rho_\mathrm{i}) \tag{19}$$

onto the tree-level logical qubit held by the photons. Here, $\circ$ indicates composition of quantum channels. Then, we define the quantum channel $\mathcal{T}_{p_0,\eta}(\rho)$ as the quantum channel implemented on the tree-level logical qubit by Steps 4 and 5. The final state is therefore

$$\rho_\mathrm{f} = \mathcal{T}_{p_0,\eta} \circ \mathcal{D}_{p_0} \circ \mathcal{D}_{p_0}(\rho_\mathrm{i}). \tag{20}$$

In order to determine $\mathcal{T}_{p_0,\eta}(\rho)$ we turn to numerical methods. We use the quantum-network simulator NetSquid [3] to estimate the Choi state [4] of $\mathcal{T}_{p_0,\eta}(\rho)$. We do so as follows. First, we prepare the Bell state $\left|\Phi^+\right\rangle = \frac{1}{\sqrt{2}}(\left|00\right\rangle + \left|11\right\rangle)$. Next, we perfectly teleport one of these two qubits onto a tree-level logical qubit (as done in Step 3). Then, we subject each of the qubits in the tree to a pure loss channel with loss probability $1-\eta$, and then to a depolarizing channel $\mathcal{D}_{p_0}$. Finally, we simulate the heralded-storage procedure. Conditioned on heralded storage being successful, the two-qubit state is now the Choi state

$$\left|\mathrm{Choi}\right\rangle = (\mathrm{Id} \otimes \mathcal{T}_{p_0,\eta})(\left|\Phi^+\right\rangle\left\langle\Phi^+\right|). \tag{21}$$

Here, Id is the identity quantum channel on a single qubit. This state fully characterizes the quantum channel.

Our simulations are probabilistic as each time a pure loss channel is applied a random number is sampled to determine if the photon is lost, and each time a depolarizing channel is applied, a random number is sampled to determine if there is an error (and if so, whether it is an X, Y or Z error). For a range of values of p_0, η and the branching vector of the tree code we have performed this procedure one million times to obtain reliable statistics. The simulation code used to obtain the data can be found in https://github.com/bernwo/code-concatenated-quantum-repeater. Both the raw and processed data can be found in https://doi.org/10.4121/b9c7327e-97b2-4ea2-9b74-18c51f265027.v1.

We investigated this for all the parameter values $\mathbf{t} \in \{[4,13,4],[5,11,4],[4,14,4],[4,12,5]\}$ found via the numerical optimization of the secret key rate, and find that

$$\left|\mathrm{Choi}\right\rangle \approx (\mathrm{Id} \otimes \mathcal{D}_{p_0})(\left|\Phi^+\right\rangle\left\langle\Phi^+\right|) \tag{22}$$

and thus

$$\mathcal{T}_{p_0,\eta} \approx \mathcal{D}_{p_0}. \tag{23}$$

This allows us to conclude

$$\rho_\mathrm{f} \approx \mathcal{D}_{p_0} \circ \mathcal{D}_{p_0} \circ \mathcal{D}_{p_0}(\rho_\mathrm{i}). \tag{24}$$

Because $\mathcal{D}_{p_1} \circ \mathcal{D}_{p_2} = \mathcal{D}_{p_1 p_2}$ we then have

$$\rho_{\mathrm{f}} \approx \mathcal{D}_{p_0^3}(\rho_{\mathrm{i}}) \equiv \mathcal{D}'_{\epsilon_{\mathrm{r}}}(\rho_{\mathrm{i}}). \tag{25}$$

Here, the last part of the equation defines the re-encoding error probability ϵ_{r}. Simply equating both sides then reveals

$$\epsilon_{\mathrm{r}} = \frac{3}{4}(1 - p_0^3) = \frac{3}{4}\left(1 - \left(\frac{4}{3}(1 - \epsilon_0)\right)^3\right) = 3\epsilon_0 + \mathcal{O}(\epsilon_0^2). \tag{26}$$

We thus finally conclude that, for a small error probability $\epsilon_0 \ll 1$,

$$\epsilon_{\mathrm{r}} \approx 3\epsilon_0. \tag{27}$$

SUPPLEMENTARY NOTE 11. COMPARISON WITH OTHER QUANTUM REPEATER SCHEMES

There exists numerous proposals for quantum repeater architectures, which all seek to lower the hardware requirements for high-rate, long-distance quantum communication [5–21]. Our protocol can be characterized as a one-way quantum repeater. This type of repeaters are particularly promising for hardware with an efficient spin-photon interface but limited memory performance both in terms of storage time and multiplexing capability.

The work of Ref. [16] showed that the use of photonic tree-cluster states in conjunction with efficient spin-photon interfaces could significantly reduce the qubit resources per repeater node compared with previous proposals. We refer the reader to the supplementary information of Ref. [16] for a detailed discussion of this aspect where comparison with both quantum emitter-based architectures [14, 22] and linear optics based approaches [23–25] has been performed.

We have summarized the comparison of our proposed quantum-repeater scheme in Supplementary Table 1 with both the original proposal in Ref. [16], the GKP-based protocols of Refs. [17, 18, 20] and a recent photonic cluster state repeater proposal [26]. From the table, it is seen that our work significantly improves on previous work by allowing for a fault-tolerant repeater with minimized qubit resources per repeater node while representing a fundamentally different route than continuous variable GKP approaches.

The key drawback of Ref. [16] is that the lack of fault-tolerance which meant that very low error rates are needed for intercontinental distances. In this work, we have shown how this can be addressed efficiently through code concatenation and flag-based quantum error correction. Importantly, we demonstrate a fully fault-tolerant architecture with only a modest increase in resources compared to the protocol of Ref. [16].

Recently, there has been a number of proposals that also exploit code-concatenation for fault-tolerant quantum repeater architectures [17–20]. These proposals focus on the combination of a GKP code with a small discrete-variable (DV) quantum error correcting code. Moreover, if one has access to almost lossless hardware, then in some configurations bare GKP qubits can be sufficient [18] and in the limit of very high squeezing further performance benefits could be achieved by considering GKP qudits [21]. These works represent a fundamentally different approach than the one we consider here. In particular, the experimental challenges are very different for such approaches compared to ours. The generation of an optical GKP state remains a daunting experimental challenge, which is very different from the arguably also challenging task of generating the large photonic cluster states that our protocol requires. We do note, however, that recent experimental achievements of the generation of multiple-photon cluster states have already demonstrated some of the key functionality required for our proposal [27, 28]. We also note the recent experimental realization of optical GKP states [29] that provides the initial push towards the feasibility of GKP-based protocols. In general, however, most of the systems currently being explored for quantum information processing are based on qubits. The DV approach that we pursue in this work, is thus applicable to a broader range of the experimental systems.

Schemes	Characteristics	Performance
Current work	Tolerance against both loss and operational errors by concatenating tree cluster states with ⟦5,1,3⟧ code. Fault-tolerance against operational error achieved via flag qubit on the ⟦5,1,3⟧ code level. Each TYPE I node requires 15 matter qubits to generate tree cluster states, while each TYPE II node requires 2 more qubits to perform error correction procedure on the outer code level. Requires classical feed forward.	Assumed operational error $\sim 0.1\%$. Two-qubit gate errors are assumed to be dominant. A secret key rate of $\sim$ 7.5 kHz assuming the six-state quantum key distribution protocol was achieved with $\eta_{\mathrm{d}} = 0.95$ at $L_{\mathrm{tot}} \approx 800$ km, optimized with respect to a cost function (see main text). Ratio of inter-repeater distance to attenuation distance found to be $L_0/L_{\mathrm{att}} \sim 0.11$ with $L_{\mathrm{att}} = 20$ km.
Niu *et al.* [26]	Based on a purely photonic approach using measurement-based CSS codes where no matter qubits are needed at the repeater nodes. Resources required for encoded-state generation are not considered. One of the CSS codes considered is the ⟦48,6,8⟧ code. Does not consider code concatenation. No classical feed forward required.	No operational errors included and only loss errors are considered. An effective transmission probability of ~ 0.8 was found for $\eta_{\mathrm{d}} = 0.95$ at $L_{\mathrm{tot}} = 1000$ km. Ratio of inter-repeater distance to attenuation distance found to be $L_0/L_{\mathrm{att}} \sim 0.12$ with $L_{\mathrm{att}} = 22$ km.
Borregaard *et al.* [16]	Loss tolerance achieved via tree-cluster states. Each node requires a quantum emitter and 2 auxiliary memory spins to generate a tree-cluster state. Does not consider code concatenation. Requires classical feed forward.	Assumed operational error $\sim 0.1\%$. A secret key rate of ~ 0.1 kHz assuming the six-state quantum key distribution protocol was achieved with $\eta_{\mathrm{d}} = 0.95$ at $L_{\mathrm{tot}} \approx$ 800 km, optimized with respect to a cost function. Ratio of inter-repeater distance to attenuation distance found to be $L_0/L_{\mathrm{att}} \sim 0.20$ with $L_{\mathrm{att}} = 20$ km.
Fukui *et al.* [18]	CV-based two-way scheme requiring optical GKP states. Repeaters generate GKP Bell pairs where each half of the Bell pair is sent in opposite direction for loss-tolerant entanglement swapping. Based on the GKP analog information from swapping one can introduce post-selection to trade-off success probability with fidelity of entanglement generation.	Secret-key rate of 0.2 secret bits per mode/per protocol run assuming the six-state quantum key distribution protocol with homodyne detection efficiency of $\eta_{\mathrm{d}} = 0.99$ and GKP squeezing of 15.4 dB at $L_{\mathrm{tot}} \approx 1000$ km. The total number of GKP qubits needed per protocol run over this entire L_{tot} is 4×10^3. Ratio of inter-repeater distance to attenuation distance found to be $L_0/L_{\mathrm{att}} \sim 0.02$ with $L_{\mathrm{att}} = 22$ km.
Rozpędek *et al.* [17]	CV-based one-way scheme requiring optical GKP states. The repeaters are assumed to be equipped with between 3 to 10 "GKP memories" that can reliably store GKP states while performing the sequence of error correction operations on them. Concatenation of GKP state with either an outer ⟦4,1,2⟧ code or ⟦7,1,3⟧ code is considered. Requires classical feed forward.	Perfect homodyne detection and two-qubit GKP gate is assumed. For the ⟦4,1,2⟧ code, a secret key rate of 0.1 bits per mode assuming the six-state quantum key distribution protocol with repeater coupling efficiency of $\eta_{\mathrm{d}} = 0.97$ and GKP squeezing of 17.9 dB at $L_{\mathrm{tot}} \approx 5000$ km, optimized with respect to a cost function. If instead ⟦7,1,3⟧ code is used, the same performance can be achieved at $L_{\mathrm{tot}} \approx$ 10000 km. Ratio of inter-repeater distance to attenuation distance found to be $L_0/L_{\mathrm{att}} \sim 0.01$ with $L_{\mathrm{att}} = 22$ km.
Rozpędek *et al.* [20]	CV-based two-way scheme requiring optical GKP states. An all-optical approach where all the GKP operations are performed fiber-based is considered, and therefore no "GKP memories" are required. The resource states for this protocol are logical Bell pairs between a GKP qubit and a code-concatenated GKP-⟦7,1,3⟧-code qubit.	A performance of 4 secret key bits per protocol run can be achieved assuming the six-state quantum key distribution protocol with homodyne detection efficiency of $\eta_{\mathrm{d}} = 0.98$ and GKP squeezing of 15.4 dB at $L_{\mathrm{tot}} \approx 5000$ km, optimized with respect to a cost function. To achieve this performance in a fully all-photonic scenario the total number of GKP qubits needed per protocol run over this entire L_{tot} is 10^7. Ratio of inter-repeater distance to attenuation distance found to be $L_0/L_{\mathrm{att}} \sim 0.05$ with $L_{\mathrm{att}} = 22$ km.

Supplementary Table 1. Comparison with quantum repeater schemes considered in Refs. [16, 17, 20, 26]. As mentioned in the main text, η_{d} is the overall repeater efficiency, L_0 is the inter-repeater distance, L_{att} is the attenuation distance, and L_{tot} is the total distance.

SUPPLEMENTARY REFERENCES

[1] R. Chao and B. W. Reichardt, Quantum error correction with only two extra qubits, Phys. Rev. Lett. **121**, 050502 (2018).
[2] V. Scarani, H. Bechmann-Pasquinucci, N. J. Cerf, M. Dušek, N. Lütkenhaus, and M. Peev, The security of practical quantum key distribution, Rev. Mod. Phys. **81**, 1301 (2009).
[3] T. Coopmans, R. Knegjens, A. Dahlberg, D. Maier, L. Nijsten, J. de Oliveira Filho, M. Papendrecht, J. Rabbie, F. Rozpędek, M. Skrzypczyk, L. Wubben, W. de Jong, D. Podareanu, A. Torres-Knoop, D. Elkouss, and S. Wehner, NetSquid, a NETwork Simulator for QUantum Information using Discrete events, Communications Physics **4**, 1 (2021).
[4] M.-D. Choi, Completely positive linear maps on complex matrices, Linear Algebra Appl. **10**, 285 (1975).
[5] J. Yin, Y. Cao, Y.-H. Li, S.-K. Liao, L. Zhang, J.-G. Ren, W.-Q. Cai, W.-Y. Liu, B. Li, H. Dai, G.-B. Li, Q.-M. Lu, Y.-H. Gong, Y. Xu, S.-L. Li, F.-Z. Li, Y.-Y. Yin, Z.-Q. Jiang, M. Li, J.-J. Jia, G. Ren, D. He, Y.-L. Zhou, X.-X. Zhang, N. Wang, X. Chang, Z.-C. Zhu, N.-L. Liu, Y.-A. Chen, C.-Y. Lu, R. Shu, C.-Z. Peng, J.-Y. Wang, and J.-W. Pan, Satellite-based entanglement distribution over 1200 kilometers, Science **356**, 1140 (2017).
[6] M. K. Bhaskar, R. Riedinger, B. Machielse, D. S. Levonian, C. T. Nguyen, E. N. Knall, H. Park, D. Englund, M. Lončar, D. D. Sukachev, and M. D. Lukin, Experimental demonstration of memory-enhanced quantum communication, Nature **580**, 60 (2020).
[7] M. Pompili, S. L. N. Hermans, S. Baier, H. K. C. Beukers, P. C. Humphreys, R. N. Schouten, R. F. L. Vermeulen, M. J. Tiggelman, L. dos Santos Martins, B. Dirkse, S. Wehner, and R. Hanson, Realization of a multinode quantum network of remote solid-state qubits, Science **372**, 259 (2021).
[8] W. J. Munro, K. Azuma, K. Tamaki, and K. Nemoto, Inside quantum repeaters, IEEE Journal of Selected Topics in Quantum Electronics **21**, 78 (2015).
[9] S. Muralidharan, L. Li, J. Kim, N. Lütkenhaus, M. D. Lukin, and L. Jiang, Optimal architectures for long distance quantum communication, Sci. Rep. **6**, 20463 (2016).
[10] H.-J. Briegel, W. Dür, J. I. Cirac, and P. Zoller, Quantum repeaters: The role of imperfect local operations in quantum communication, Phys. Rev. Lett. **81**, 5932 (1998).
[11] L. M. Duan, M. D. Lukin, J. I. Cirac, and P. Zoller, Long-distance quantum communication with atomic ensembles and linear optics, Nature **414**, 413 (2001).
[12] N. Sangouard, C. Simon, H. de Riedmatten, and N. Gisin, Quantum repeaters based on atomic ensembles and linear optics, Rev. Mod. Phys. **83**, 33 (2011).
[13] W. J. Munro, A. M. Stephens, S. J. Devitt, K. A. Harrison, and K. Nemoto, Quantum communication without the necessity of quantum memories, Nat. Photonics **6**, 777 (2012).
[14] S. Muralidharan, J. Kim, N. Lütkenhaus, M. D. Lukin, and L. Jiang, Ultrafast and fault-tolerant quantum communication across long distances, Phys. Rev. Lett. **112**, 250501 (2014).
[15] F. Ewert, M. Bergmann, and P. van Loock, Ultrafast long-distance quantum communication with static linear optics, Phys. Rev. Lett. **117**, 210501 (2016).
[16] J. Borregaard, H. Pichler, T. Schröder, M. D. Lukin, P. Lodahl, and A. S. Sørensen, One-way quantum repeater based on near-deterministic photon-emitter interfaces, Phys. Rev. X **10**, 021071 (2020).
[17] F. Rozpędek, K. Noh, Q. Xu, S. Guha, and L. Jiang, Quantum repeaters based on concatenated bosonic and discrete-variable quantum codes, npj Quantum Inf. **7**, 102 (2021).
[18] K. Fukui, R. N. Alexander, and P. van Loock, All-optical long-distance quantum communication with gottesman-kitaev-preskill qubits, Phys. Rev. Research **3**, 033118 (2021).
[19] F. Schmidt and P. van Loock, Quantum error correction with higher gottesman-kitaev-preskill codes: Minimal measurements and linear optics, Phys. Rev. A **105**, 042427 (2022).
[20] F. Rozpędek, K. P. Seshadreesan, P. Polakos, L. Jiang, and S. Guha, All-photonic multiplexed quantum repeaters based on concatenated bosonic and discrete-variable quantum codes, arXiv preprint arXiv:2303.14923 (2023).
[21] F. Schmidt, D. Miller, and P. van Loock, Error-corrected quantum repeaters with gkp qudits, arXiv preprint arXiv:2303.16034 (2023).
[22] A. N. Glaudell, E. Waks, and J. M. Taylor, Serialized quantum error correction protocol for high-bandwidth quantum repeaters, New J. Phys. **18**, 093008 (2016).
[23] K. Azuma, K. Tamaki, and H.-K. Lo, All-photonic quantum repeaters, Nat. Commun. **6**, 10.1038/ncomms7787 (2015).
[24] F. Ewert and P. van Loock, Ultrafast fault-tolerant long-distance quantum communication with static linear optics, Phys. Rev. A **95**, 012327 (2017).
[25] S.-W. Lee, T. C. Ralph, and H. Jeong, Fundamental building block for all-optical scalable quantum networks, Phys. Rev. A **100**, 052303 (2019).
[26] D. Niu, Y. Zhang, A. Shabani, and H. Shapourian, All-photonic one-way quantum repeaters, arXiv preprint arXiv:2210.10071 (2022).
[27] P. Thomas, L. Ruscio, O. Morin, and G. Rempe, Efficient generation of entangled multiphoton graph states from a single atom, Nature **608**, 677 (2022).
[28] D. Cogan, Z.-E. Su, O. Kenneth, and D. Gershoni, Deterministic generation of indistinguishable photons in a cluster state, Nat. Photonics **17**, 324 (2023).
[29] S. Konno, W. Asavanant, F. Hanamura, H. Nagayoshi, K. Fukui, A. Sakaguchi, R. Ide, F. China, M. Yabuno, S. Miki, H. Terai, K. Takase, M. Endo, P. Marek, R. Filip, P. van Loock, and A. Furusawa, Propagating gottesman-kitaev-preskill states encoded in an optical oscillator, arXiv preprint arXiv:2309.02306 (2023).
